# Selenium nanoparticles coated bacterial polysaccharide with potent antimicrobial and anti-lung cancer activities

**DOI:** 10.1038/s41598-023-48921-9

**Published:** 2023-12-10

**Authors:** Nourhan S. Shehata, Bassma H. Elwakil, Salma S. Elshewemi, Doaa A. Ghareeb, Zakia A. Olama

**Affiliations:** 1 Department of Medical Laboratory Technology, Faculty of Applied Health Sciences Technology, Pharos University in Alexandria, Alexandria, Egypt; 2 Department of Botany and Microbiology, Faculty of Science, Alexandria University, Alexandria, Egypt; 3 Zoology Department, Faculty of Science, Alexandria University, Alexandria, Egypt; 4 Biological Screening and Preclinical Trial Lab, Biochemistry Department, Faculty of Science, Alexandria University, Alexandria, 21526 Egypt

**Keywords:** Nanobiotechnology, Cancer, Cancer therapy, Infectious diseases

## Abstract

Bacterial exopolysaccharides are homopolymeric or heteropolymeric polysaccharides with large molecular weights (10–1000 kDa). Exopolysaccharides' functional uses and potential have revolutionized the industrial and medicinal industries. Hence, the aim of the present study was to optimize the production of bacterial exopolysaccharide and apply it as a capping agent for selenium nanoparticles synthesis. Exopolysaccharide (EPS) producing Lactic acid bacteria (LAB) were isolated from dairy products then biochemically characterized and assessed for their potential antimicrobial effect. The most potent EPS producer was identified as *Lactiplantibacillus plantarum* strain A2 with accession number OP218384 using 16S rRNA sequencing. Overall, FTIR data of the extracted EPS revealed similarity with amylopectin spectrum. ^1^H NMR spectrum revealed an α-anomeric configuration of the glycosidic linkage pattern in the polysaccharides while the ^13^C NMR spectrum can also be separated into two main portions, the anomeric carbons region (δ 98–102 ppm) and the non-anomeric carbons region (δ 60–81 ppm). Antimicrobial activity of the produced EPS showed maximum activity against *Staphylococcus aureus*, MRSA, *Enterobacter aerogenes, Klebsiella pneumoniae* and *Candida albicans* respectively. The EPS capsule layer surrounding the bacterial cells was detected by TEM study. Optimization of EPS production was evaluated using Taguchi design, trial 23 reported the highest biomass yield and EPS output (6.5 and 27.12 g/L respectively) with 2.4 and 3.3 folds increase (from the basal media) respectively. The optimized exopolysaccharide was used as a capping and stabilizing agent for selenium nanoparticles (EPS-SeNPs) synthesis. Zeta potential, size and PDI of the synthesized nanoparticles were − 19.7 mV, 45–65 nm and 0.446 respectively with strong bactericidal and fungicidal effect against the tested pathogens. Complete microbial growth eradication was recorded after 6, 8 and 10 h against *Staphylococcus aureus*, *Candida albicans* and *Klebsiella pneumoniae* respectively. EPS-SeNPs showed a potent antioxidant effect reached 97.4% and anticancer effect against A549 lung cancer cell line (IC_50_ reached 5.324 µg/mL). EPS-SeNPs inhibited cancerous cell growth at S phase. Moreover, molecular studies revealed the anti-apoptotic activity of Bcl2's was inhibited and Bax was activated. The present investigation successfully synthesized selenium nanoparticles through bacterial EPS with significantly high antimicrobial and anticancer activity.

## Introduction

Respiratory infections are among the leading causes of death worldwide, especially in patients with lung cancer. Recently, the prevalence of microorganisms that are multi-drug resistant (MDR) has increased which limited the possible treatment options. In the instance of respiratory infections, the most prevalent MDR microorganisms were *Klebsiella pneumoniae*, *Escherichia coli*, *Pseudomonas aeruginosa* and *Staphylococcus aureus*^1,2^.

On the other hand, cancer is one of the most fatal diseases, which can be controlled by genetic, environmental, and pharmacological variables^3^. The major leading cause of cancer-related mortality in both men and women worldwide reported by the World Health Organization (WHO) was lung cancer^4^. The most prevalent histological form of lung cancer is the adenocarcinoma subtype of non-small-cell lung cancer (NSCLC)^4,5^. Currently, lung cancer treatments have several side effects that could have a severe influence on the patients^6^. Several infectious agents have been related to the increased incidence of cancer with the concept that pathogenic microorganisms were responsible for 16.1% of all cancers^7^.

Bacterial polysaccharide is a biological macromolecule with numerous biological functions^8^. EPS was reported to have antioxidant, anticancer, anti-diabetic, and immunomodulatory activities^9^. Moreover, bacterial polysaccharide has been applied as reducing and coating agent for the biological synthesis of metallic nanoparticles^9,10^. Lactic acid bacteria (LAB) are the most commonly used safe probiotics with high EPS content^11,12^. One of the most adaptable and promising species within the LAB group is *Lactobacillus plantarum*, which has lately been referred to as *Lactiplantibacillus* (Lpb.) *plantarum* subsp. *plantarum*^13^. *Lactobacillus plantarum* is a bacterium that exhibits a Gram-positive staining with straight rod shape. The cell wall is composed of either ribitol- or glycerol- teichoic acid type, however some strains have an atypical teichoic acid composition. The cell wall's peptidoglycan is classified as the meso-diaminopimelic acid (DAP) type. *Lactiplantibacillus plantarum* strains are often found in several environments, including the gastrointestinal system, feces, fermented foods, and plants, among other niches^14^.

Bacterial polysaccharide is a water-soluble polymer with minimal toxicity and has been employed as a surface coating for selenium nanoparticles (SeNPs). Selenium is a trace element that performs a variety of biological functions in human health via selenium-containing enzymes and anti-oxidant actions including free radicals inhibition such as 2,2-diphenyl-1-picrylhydrazyl (DPPH)^15^. SeNPs have high bioavailability and less toxicity compared to selenium itself which paved the way to use SeNPs in wide range of therapeutic applications^10,15^. Selenium nanoparticles coated with bacterial polysaccharide could greatly hinder the microbial and cellular melanoma growth^16^. The present work aimed to evaluate the bacterial polysaccharides nanoparticles activity against MDR bacteria and cancerous cell lines in vitro as a promising tool for biomedical applications.

## Materials and methods

### Microorganisms

*Lactiplantibacillus plantarum* strain A2 was isolated from dairy products as polysaccharide producer and was identified biochemically and through 16SrRNA. Some pathogens were used in the present work namely: *Escherichia coli*, *Pseudomonas aeruginosa*, *Enterobacter aerogenes*, *Staphylococcus aureus*, methicillin resistant *Staphylococcus aureus* (MRSA), *Klebsiella pneumoniae*, *Proteus vulgaris* and *Candida albicans* which were kindly provided and identified using VITEK (BIOMERIEUX, USA) by the Surveillance Microbiology Department of Pediatric Al-Shatby Hospital, Alexandria.

### Sample collection and bacterial isolation

Different dairy product sources namely: buffalo, cow raw milk and commercial yogurt were all purchased from local markets for lactic acid bacteria (LAB) isolation. One (1) g of yogurt was homogenized in 10 mL phosphate buffer solution (PBS) pH 6.5. Serial dilutions (10 to 10^–10^) of each homogenized sample were prepared, plated on MRS agar supplemented with 10% sucrose^17,18^ and incubated at 37 °C for 48 h. Morphologically different colonies were selected and purified then examined for its ropy characteristics^19^. The pure cultures were kept at − 20 °C in MRS broth supplemented with 28% glycerol (v/v)^20^ with monthly transfer on fresh media.

### Phenotypic characterization of polysaccharide producing LAB

The selected pure colonies were phenotypically identified^20^ and each Gram-positive bacilli, catalase and coagulase-negative isolate was chosen for further work. The ropy nature of EPS-producing isolates was identified by the loop touch method according to Nambiar et al.^21^. Exopolysaccharide production was confirmed by culturing the selected isolates on Congo red agar (CRA) as a primary screening method, which allowed the detection of exopolysaccharide producing bacteria through staining the bacterial colonies black^22^.

#### Simulated gastric juice survivability test

The tolerance to simulated gastrointestinal tract conditions were assessed according to Zhang et al.^23^.The simulated gastric juice was obtained by adding 0.3 g of pepsin to 100 mL of 0.9% sterile saline, then adjusting the pH to 3.0 using 1 M HCl. While the simulated intestinal juice solution was prepared by dissolving 0.2 g of trypsin and 0.3 g of ox-bile salts in 100 mL of sterile saline with a concentration of 0.9%. Then the pH of the solution was adjusted to 8.0 using 1 M NaOH. Ten (10) mL of each LAB isolates fresh culture were centrifuged for 10 min at 8000×*g* at room temperature. The pellets were then washed with an equal volume of sterile normal saline then recentrifuged. The collected cells were resuspended in 10 mL of simulated gastric and intestinal juice one at a time and incubated at 37 °C for 3 h and 7 h respectively.

1 $$\mathrm{Survival\, rate }(\mathrm{\%})=({\text{N}}1/{\text{N}}0)\times 100$$

in which N0 is the number of viable bacteria at 0 h (CFU/mL) and N1 is the number of viable bacteria in artificial gastrointestinal fluid at 3 or 7 h (CFU/mL).

#### Blood hemolysis (safety evaluation)

The blood hemolytic activity of EPS producers was assessed by inoculating the isolates onto blood agar plates containing 5.0% sheep blood. After 24 h of incubation, the plates were checked for beta, alpha, or gamma hemolysis^24^.

#### Extraction and purification of EPS

The selected isolates were inoculated and incubated in MRS broth at 37 °C for 24 h, then after incubation the bacterial cultures were centrifuged at 8000×*g*, for 20 min at 4 °C. Trichloroacetic acid (TCA) was added to the supernatant (cell-free supernatant (CFS)) drop wise for protein removal with continues stirring for 30 min at 90 rpm followed by centrifugation (at 8000×*g*, for 20 min, 4 °C). The supernatant was treated with absolute cold ethanol (2:1 v/v) and stored over night at 4 °C for EPS precipitation^12,17^. The resulting precipitate was collected by centrifugation (8000×*g* for 20 min), the pellets were washed with deionized water and lyophilized using Freeze Dryer (ILSHIN BIOBASE, America/human lab instrument vacuum freeze dryer FDI- 0650 Korea).

#### Antimicrobial activity of exopolysaccharides

Antimicrobial activity of the obtained crude EPS was achieved using disc diffusion method^25,26^. One hundred (100) μL of the pathogen’s suspension (1.5 × 10^6^ CFU/mL) (0.5 McFarland) were spread on Muller Hinton agar (MHA) plates, then sterile discs were loaded with 25 μL of each extracted EPS one at time and placed on the inoculated MHA plates. After 18 h of incubation at 37 °C, results were recorded as inhibition zone diameter (mm)^25,27^.

#### Probiotic properties of *Lactobacillus* isolates

The potent EPS producer with promising antimicrobial activity were tested for their probiotic tolerance properties against different acid and bile salts according to Nath et al.^11^ and Yadav et al.^24^ respectively.

### Bacterial identification

16S rRNA sequencing was used to identify the most promising EPS producing bacteria with the highest antimicrobial activity (Isolate no.1). The universal primers 27F (5-AGAGTTTGATCMTGGCTCAG) and 1492R (5-TACCTTG TTACGACTT) were used to amplify the 16 s rRNA gene from isolated genomic DNA. Multiple sequence alignment was performed after 16S rDNA sequencing using the National Center for Biotechnology Information (NCBI) database. Finally, the phylogenetic tree was assembled via distance matrix analysis using the NT system for the promising isolate.

#### Chemical composition of EPS

##### Protein and total sugar content determination

Protein content was estimated by Bradford method^28,29^ with bovine serum albumin as a standard^12,30^.

#### Characterization and identification of EPS

Fourier transform infrared spectroscopy (FT-IR) analysis.

Fourier-transform infrared spectroscopy was used to determine the functional groups of the extracted EPS using KBr method within spectrum range from 400 to 4000 cm^−1^ using Benchtop Cary 630 FTIR spectrometer.

##### Nuclear magnetic resonance (NMR) spectroscopy analysis

In order to assess the structure and determine the conformation of polysaccharides, NMR spectroscopy was used^31^. NMR analysis for EPS was done via Bruker High Performance; Digital FT-NMR Spectrometer Avance III 400 MHz, Switzerland. 10 and 30 mg/mL EPS was dissolved in D_2_O for ^1^H NMR and ^13^C NMR respectively^32^.

##### LC–ESI–MS (liquid chromatography electrospray ionization tandem mass spectrometric)

XEVO TQD triple quadruple instrument was used with the ESI–MS positive and negative ion acquisition modes. Autosampler injector (Switzerland), a mass spectrometer, and Waters Corporation (Milford, MA01757, U.S.A) were used. ACQUITY UPLC-BEH C18 column (1.7 µm–2.1 × 50 mm) was used with gradient mobile phase at a flow rate of 0.2 mL/min which consisted of two eluents: eluent A (H_2_O acidified with 0.1% formic acid), and eluent B (acetonitrile acidified with 0.1% formic acid). The peaks and spectra were analyzed with Maslynx 4.1 software.

##### Transmission electron microscopic (TEM) study of the potent EPS producing strain

Cell morphology and ultrastructure of the potent strain were examined using TEM (JEM-100 CX Joel, USA with a resolution of 3 nm at 30kv).

### Optimization of biomass production and EPS

Optimization of the environmental and nutritional factors for maximum biomass and polysaccharide production were performed according to Taguchi array design L27 (3^8^) of experimental methodology where L is Latin square array and 27 is number of experimental runs. L27 orthogonal design was used to study 8 variables (X1 to X8) at different levels (1, 2, and 3) (Table 1).

**Table 1:** Taguchi array design for variable levels.

Independent variables	Levels			
Factors		1	2	3
Peptone (g/L)	X1	5.0	10.0	15.0
Yeast extract (g/L)	X2	3.0	5.0	7.0
Dextrose (g/L)	X3	15.0	20.0	25.0
Sucrose (g/L)	X4	15.0	20.0	25.0
pH	X5	6.0	6.5	7.0
Temp (°C)	X6	27.0	30.0	33.0
Inoculum size (mL)	X7	0.5	1.0	2.0
Culture volume (mL)	X8	25.0	50.0	75.0

Data processing was evaluated by S/N ratio (the ratio of the target value to the deviation from its mean). In Taguchi design, the target value (mean) represents the signal, and the standard deviation for the response variable represents Noise. For calculating the S/N ratio, the larger-the-better quality characteristic was selected. S/N ratio was calculated according to the following equation: (Eq. 2)

2 $$ {\text{S}}/{\text{N}}\;({\text{larger}}\;{\text{is}}\;{\text{better}}) = - 10*\log \left( {\sum (1/Y^{2} )} \right) {\mathinner{\mkern2mu\raise1pt\hbox{.}\mkern2mu \raise4pt\hbox{.}\mkern2mu\raise7pt\hbox{.}\mkern1mu}} {\text{n}} $$

### Preparation of selenium nanoparticles EPS-SeNPs

Green synthesis of selenium nanoparticles coated with EPS (EPS-SeNPs) was prepared by adding 10 mM sodium selenite (Na_2_SeO_3_) as a precursor to an equal volume of EPS solution (1 mg/mL) under stirring condition at 25 °C for 30 min. Then, 40 mM of freshly prepared ascorbic acid was added drop wise into the mixture with continuous stirring at 40 °C in the dark for 4 h until the color changed from colorless to light orange. EPS-SeNPs were separated by centrifugation at 14,000 rpm for 20 min at room temperature, freeze-dried, and then stored for further analysis^33–35^.

#### Characterization of SeNPs

Zeta potential, particle size (PS), and polydispersity index (PDI) of the synthesized nanoparticles were determined using DLS analysis (Malvern Zeta sizer) according to Elnaggar et al.^26^. The ultraviolet–visible spectroscopy (UV–Vis) absorption spectrum was measured using a Shimadzu UV-1800 UV spectrophotometer in the wavelength range of 190–600 nm at 25 °C. FTIR spectrum of the synthesized nanoparticles was analyzed using KBr method within spectrum ranged from 400 to 4000 cm^−1^ using FT-IR spectrophotometer (Agilent technologies; Benchtop Cary 630 FTIR spectrometer, Malaysia). The structure, size and shape of the synthesized EPS-SeNPs were examined using TEM (JEM-100 CX, JOEL, USA, at resolution 3 nm at 30 kV)^36^. The elemental composition percentage of the EPS-SeNPs was determined using energy dispersive X-ray spectroscopy (EDX, x-max50, an Oxford instrument EDX energy dispersive x-ray).

#### Antimicrobial activity of EPS-SeNPs

Antimicrobial activity of the synthesized EPS-SeNPs was evaluated using disc diffusion method and minimum inhibitory concentration (MIC)^37,38^. All data are the means of three trials.

### In vitro antioxidants and anticancer activities

#### Antioxidant activity

1,1-diphenyl-2-picrylhydrazyl (DPPH) antioxidant assay kit (K2078-100 Colorimetric, bio vision Inc., abcam, USA) was used to test the free radical scavenging activity of EPS and EPS-SeNPs. In 96-well plates, 100 µL of EPS and EPS-SeNPs one at a time with different concentrations were added to 100 µL of DPPH solution (4 mg DPPH dissolved in methanol). In control well, 100 µL of DPPH solution was mixed with 100 µL methanol. The mixture was vigorously stirred before being incubated at room temperature in the dark for 30 min. Finally, the absorbance was recorded at 595 nm wavelength^27^. Synthetic antioxidants (Trolox) in various doses were employed to validate the process.

The degree of scavenging was calculated according to the following equation: (Eq. 3)

3 $$ {\text{Scavenging}}\;{\text{effect}}\;(\% ) = \frac{{{\text{control}}\;{\text{absorbance}} - {\text{sample}}\;{\text{absorbance}}}}{{{\text{control}}\;{\text{absorbance}}}} \times 100 $$

The tested concentration that reported 50% inhibition (EC_50_) was measured, showing the scavenging effect percentage versus the varied concentrations.

#### Cell culture and cytotoxicity of EPS and EPS-SeNPs

The human lung adenocarcinoma cell line (A549), and normal lung fibroblasts (WI38) were obtained from the American Type Culture Collection (ATCC). Cells were cultured using DMEM (Invitrogen/Life Technologies, USA) supplemented with 10% FBS (Hyclone, USA). Cytotoxicity of EPS and EPS-SeNPs at different concentrations (100, 25, 6.3, 1.6 and 0.4 µg/mL) in comparison to Staurosporine (reference drug) was carried out for 48 h at 37 ºC in humidified atmosphere (5% CO_2_), the viable cell count was measured using MTT assay^16^. The absorbance was recorded at 450 nm via the microplate reader (BIOLINE Diagnostic LLP, BDR-206, Delhi, India). IC_50_ of each compound was calculated^39^.

Moreover, the Selectivity index was calculated as follows (Eq. 4).

4 $$\mathrm{Selectivity index}=\frac{{\text{IC}}50\,\mathrm{of\, normal\, cells }}{{\text{IC}}50\,\mathrm{of\, cancer\, cells}}$$

#### Determination of cellular reactive oxygen species (ROS)

Different EPS and EPS-SeNPs concentrations were incubated with A549 (cancerous lung cell line) and WI38 (normal lung cell line) for 72 h. Cellular ROS (Invitrogen kit, Thermo Fisher Scientific Inc., USA) levels were measured using flow cytometry in the FITC (Fluorescein isothiocyanate) channel. Briefly, the cellular ROS level was quantified by adding 100 μL of ROS assay stain solution to 1 mL of cells and 1 mL of ROS assay buffer, then incubated for 60 min at 37 °C in 5% CO_2_. After staining, cells were fixed with IC fixation buffer (cat. 00-8222, USA) and stored at 8 °C in the dark for further analysis using a flow cytometer (ROBONIK P2000 ELISA READER, India) at 450 nm.

#### Relative change in the genetic expression of proapoptotic (Caspase-3 and BAX) and anti-apoptotic (Bcl2) Genes

Total RNA was extracted from the control, EPS and EPS-SeNPs treated A549 cells. Then cDNA was synthesized using Qiagen RNA extraction/Bio-Rad syber green PCR master mix (Bio-Rad lab. Inc, Germany) and specific primers (Forward and Reverse) were: 5′- ATGTTTTCTGACGGCAACTTC -3/5′- AGTCCAATGTCCAGCCCAT -3′, 5′- ATGTGTGTGGAGACCGTCAA -3/5′- GCCGTACAGTTCCACAAAGG -3′, 5′- TGTTTGTGTGCTTCTGAGCC-3′/5′- CACGCCATGTCATCATCAAC-3′ and 5′-ATC GTG GGG CGC CCC AGG CAC-3′/5′-CTC CTT AAT GTC ACG CAC GAT TTC-3′ for BAX, Bcl2, Caspase-3 and β-actin genes, respectively. Rotor-Gene 6000 Series Software 1.7 Build 87 was used. The relative gene expression was calculated by using the rule of 2^−∆∆CT^^40^.

#### Cell cycle analysis assay

Cell apoptosis was measured using Annexin Propidium Iodide V-FITC/PI apoptosis detection kit (ab139418, USA)^41^. The harvested A549 cells (1 × 10^6^) were fixed in 66% ethanol and stored at 4 °C for 2 h. Then the cells were rehydrated in PBS and stained with propidium iodide and RNase for 30 min. Finally, Propidium iodide fluorescence intensity was measured on FL2 of a flow cytometer with 488 nm laser excitation (Excitation maximum, 493 nm; Emission maximum, 636 nm), then the data were analyzed by a flow cytometer (BD FAC SC alibur, BD Biosciences, Canada).

### Statistical analysis

All statistical analyses were performed using Minitab 19 software. Data processing was done by estimating the S/N ratio. In the Taguchi design, the target value (mean) represents the signal, Analysis of Variance (ANOVA) at the 0.05 significance level.

### Ethical approval

This research work was approved for publication approved by the Institutional Review Board of faculty of science, Alexandria University; approval number: AU/04/23/04/27/637.

## Results

### Isolation of EPS producing lactic acid bacteria (LAB)

Different raw milk and yogurt samples were screened for the presence of LAB. Ten (10) morphologically different isolates were selected for further analyses. Based on the distinct characteristics of LAB (Table 2), Gram-positive bacilli, catalase and coagulase-negative isolates were selected for further analyses^42^. The slimy appearance on MRS plates (Fig. S1) and the reaction on Congo red agar also confirmed the exopolysaccharide production as reported by Patil et al.^43^. To be efficient LAB, a probiotic must withstand the acidic conditions of the stomach, and then tolerate exposure to bile acids in the small intestine^44^. The survival rate of LAB in the gastrointestinal juice was presented in Table 3. Data revealed that highest survival rates in gastric juice (pH 3.0) and intestinal juice (pH 8.0) was recorded for isolate number 1 (97.87 and 86.84% respectively).

**Table 2:** Morphological and biochemical characteristics of the isolated LAB.

Isolate no	Source	Colony morphology	Gram Stain	Cell shape	Biochemical parameter		Color of colony on Congo red agar	Growth at different pH			Growth at different Bile salt %		
					Catalase activity	Coagulase activity		2	3	4	0.3	1	2
1	Raw buffalo milk	Entire, circular white to creamy color	Gram positive	Bacilli	Catalase negative	Coagulase negative	Black	+	+	+	+	+	+
2		Entire, circular white to creamy color	Gram positive	Bacilli	Catalase negative	Coagulase negative	Black	+	+	+	+	+	+
3		Entire, circular white to creamy color	Gram positive	Bacilli	Catalase negative	Coagulase negative	Black	+	+	+	+	+	+
4		Entire, circular white to creamy color	Gram positive	Bacilli	Catalase negative	Coagulase negative	Dark red	**+**	**+**	**+**	**+**	**+**	**+**
5	Raw caw milk	Entire, circular white to creamy color	Gram positive	Bacilli	Catalase negative	Coagulase negative	Black	+	+	+	+	+	+
6		Entire, circular white to creamy color	Gram positive	Coccobacilli	Catalase negative	Coagulase negative	Black	+	+	+	+	+	+
7		Entire, circular white to creamy color	Gram positive	Bacilli	Catalase negative	Coagulase negative	Black	+	+	+	+	+	+
8		Entire, circular white to creamy color	Gram positive	Short bacilli	Catalase negative	Coagulase negative	Dark red	+	+	+	+	+	+
9	Yogurt	Entire, circular white to creamy color	Gram positive	Short bacilli	Catalase negative	Coagulase negative	Dark red	+	+	+	+	+	+
10		Entire, circular white to creamy color	Gram positive	Coccobacilli	Catalase negative	Coagulase negative	Dark red	+	+	+	+	+	+

**Table 3:** Effect of gastrointestinal juice on the LAB survival rate.

Isolate no	Initial concentration at 0 h	Stimulated gastric juice at pH 3.0		Stimulated intestinal juice at pH 8.0	
	(log10 CFU/mL)	3 h (log10 CFU/mL)	Survival rate (%)	7 h (log10 CFU/mL)	Survival rate (%)
1	9.22 ± 0.02	9.02 ± 0.05	97.87	8.01 ± 0.02	86.84
2	8.77 ± 0.02	7.96 ± 0.07	90.80	6.21 ± 0.02	70.85
3	8.94 ± 0.04	8.42 ± 0.03	94.15	7.20 ± 0.03	80.50
4	9.11 ± 0.03	8.78 ± 0.03	96.38	7.12 ± 0.04	78.09
5	8.86 ± 0.02	7.18 ± 0.03	81.05	6.11 ± 0.05	68.97
6	9.20 ± 0.02	8.18 ± 0.03	88.95	7.12 ± 0.06	77.46
7	8.91 ± 0.04	8.32 ± 0.02	93.41	7.97 ± 0.02	89.45
8	9.06 ± 0.02	7.95 ± 0.04	87.78	6.15 ± 0.05	67.94
9	9.29 ± 0.04	8.49 ± 0.01	91.32	7.50 ± 0.02	80.67
10	9.46 ± 0.05	8.31 ± 0.02	87.91	7.53 ± 0.02	79.56

#### Screening for exopolysaccharide production

**Figure 1:**
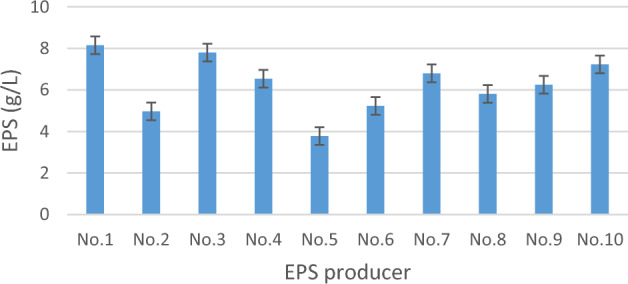
EPS production by the isolated EPS producing LAB.

Crude EPS of all the isolates under test were extracted by ethanol precipitation technique. It was noticed that LAB’s EPS dry weight ranged from 3.78 to 8.15 g/L (Fig. 1). Torino et al.^45^, reported that *L. reuteri* Lb121 EPS dry weight was 10 g/L. On the other hand, Nguyen et al.^8^ mentioned that the highest EPS dry weights yield was noticed when LAB isolates were inoculated on 20% sucrose (as a sole carbon source). Yu et al.^46^ revealed that LAB isolated from kimchi produced up to 9.80 g/L of EPS in response to the high sucrose concentration in the fermentation media.

### Screening for the antimicrobial activity of the extracted EPS

Antimicrobial activity of the extracted EPS was evaluated by disc diffusion method. Isolate no.1 was the most promising EPS producing LAB with the highest antimicrobial activity against most of the tested pathogens, especially *Staphylococcus aureus*, MRSA, *Enterobacter aerogenes, Klebsiella pneumoniae* and *Candida albicans* with inhibition zone diameter (IZ) reached 8, 8, 8, 7 and 8 mm respectively (Table 4).

**Table 4:** Antimicrobial activity of the Extracted EPS against the tested pathogens.

Tested pathogens	Inhibition zone diameter (mm)						
	*E. coli*	*Ent. aerogenes*	*S. aureus*	*K. pneumoniae*	*P. vulgaris*	MRSA	*Candida albicans*
Isolate no.1	5.5	8.0	8.0	7.0	R	8.0	8.0
Isolate no.2	5.5	7.0	6.0	7.0	R	6.0	R
Isolate no.3	5.5	6.0	6.0	6.0	R	7.0	R
Isolate no.4	6.0	5.5	R	6.0	R	8.0	6.0
Isolate no.5	5.5	6.0	6.0	5.5	R	6.0	R
Isolate no.6	R	R	6.0	6.0	6.0	6.0	R
Isolate no.7	5.5	R	6.0	6.0	R	R	5.5
Isolate no.8	R	7.0	R	R	7.0	5.5	5.5
Isolate no.9	R	7.0	R	R	6.0	7.0	6.0
Isolate no.10	5.5	8.0	R	6.0	R	8.0	R

*R* resistant

Aullybux et al.^47^ tested the antibacterial activity of EPS (extracted from *Halomonas sp.*) which exhibited promising antibacterial properties against several human pathogens namely *Enterobacter aerogenes*, *Escherichia coli*, *Proteus vulgaris* and *Staphylococcus aureus*. Similarly Saleem et al.^48^ and Hashem and Salem^37^ isolated LAB strains from traditional Chinese cheese with potent antibacterial activity against both Gram positive and negative bacteria. It is well known that the antimicrobial activity of *L. plantarum* is mainly exerted by bacteriocins (~ 60% of the reported strains) or partially characterized proteinaceous compounds, followed by organic acids or acidic conditions, and biosurfactants (BS) such as glycoproteins and EPS^49^. Broadly, the mechanism of action of these bioactive compounds is disruption and/or perforation of target cell membranes^44^.

### Identification of the most promising isolate

**Figure 2:**
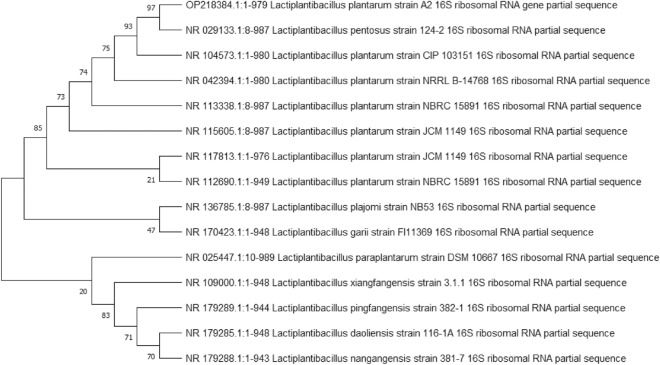
Phylogenetic tree of the most potent isolate.

16S rRNA sequencing was used to identify the most potent isolate (isolate no.1). Multiple sequence alignment was performed in accordance with the National Center for Biotechnology Information (NCBI) database. The promising strain was identified as *Lactiplantibacillus plantarum* strain A2 with accession number OP218384 (Fig. 2).

#### Protein and total carbohydrate content of the selected EPS.

The protein content of *Lactiplantibacillus plantarum* OP218384 crude EPS was 3.71 ± 0.55 mg/g, while total carbohydrate content ranged from 68.963 to 76.879 mg/g.

### Characterization of EPS

#### Fourier transform infrared spectroscopy (FT-IR) spectra analysis

**Figure 3:**
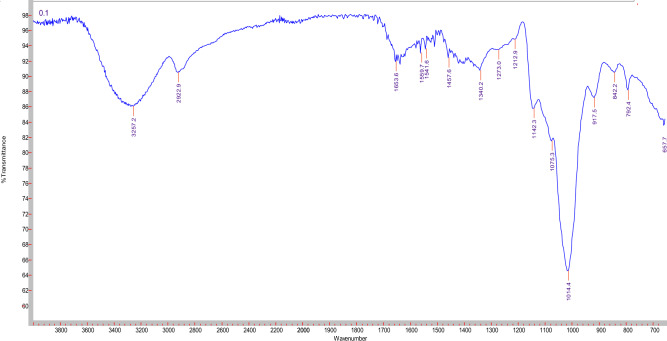
FTIR analysis of crude EPS.

The common characteristic absorption peaks of polysaccharides are the stretching vibrations of O–H bond (3200–3500 cm^−1^), C–H bond of the methylene groups (2930 cm^−1^), and saccharides ether linkage (1000–1200 cm^−1^). FTIR spectroscopic analysis of EPS (Fig. 3) revealed the broad characteristic absorption peak around 3257.2 cm^−1^, which is attributed to stretching vibrations of OH groups of the polysaccharide and the water constitutional molecules. The absorption peaks at ca. 2922.9 cm^−1^ and 1653.6 cm^−1^ signifies respectively the stretching and bending vibrations of C–H bond of methylene (CH_2_) group. In the critical identification region of polysaccharides, 1000–1200 cm^−1^, the stretching vibration band for C–O–C bond of the sugar cyclic form appeared at 1142.30 cm^−1^. The sharp weak absorption peak at approximately 917.5 cm^−1^ can be assigned to the skeletal mode vibrations of α-(1 → 4) glycosidic linkages. Whereas the presence of α-(1 → 6) glycosidic linkages can be indicated by the sharp strong absorption band at ca. 1014.4 cm^−1^. Overall, FTIR data of EPS in the functional groups region revealed similarity with amylopectin spectrum, in agreement with Miao et al.^50^.

#### Nuclear magnetic resonance (NMR) spectroscopy analysis

**Figure 4:**
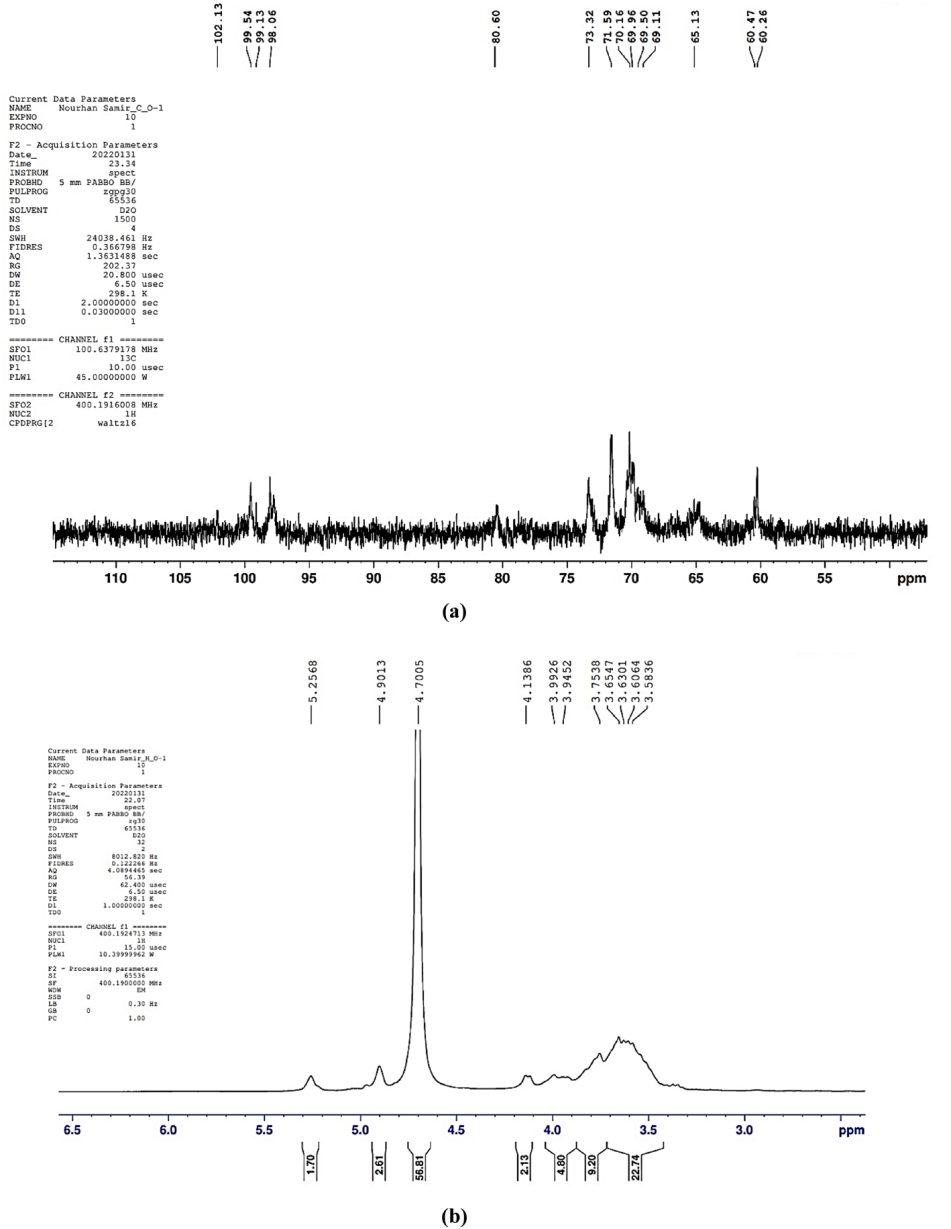
^1^H NMR (**a**) and ^13^C NMR (**b**) spectra of *Lactiplantibacillus plantarum* OP218384 EPS.

EPS of *Lactiplantibacillus plantarum* OP218384, was further analyzed using NMR spectroscopy. ^1^H NMR and ^13^C NMR analyses are usually employed for identifying the configuration of the glycosidic bond of EPS and revealing its composition^19,48^. In particular, signals appearing in the anomeric region (δ 4.5–5.5 ppm) of ^1^H NMR spectrum can differentiate between α- and β-anomeric protons of sugar residues in polysaccharides. Chemical shifts (δ) between 4.4 and 4.8 ppm represent the β-anomeric protons, whereas anomeric protons of α-linked residues usually resonate between 4.9 and 5.3 ppm^19^. In the present study, the ^1^H NMR spectrum (Fig. 4) revealed an α-anomeric configuration of the glycosidic linkage pattern in the polysaccharides. As displayed in Fig. 4, two main regions can be detected in the ^1^H NMR: the first is the anomeric protons region as characterized by the signals between δ 4.8–5.4 ppm, while the signals nearby δ 3.4–4.2 ppm signify the second region of protons linked to C2–C6. In the anomeric proton’s regions, two major peaks can be distinguished at δ 5.25 ppm and δ 4.90 ppm representing α-(1 → 4) and α-(1 → 6) linked D-glucans, respectively. The observed integration of the two peaks suggests a ratio of α-(1 → 6) linkages to α-(1 → 4) linkages of approximately 2:1. The ^13^C NMR spectrum (Fig. 4) can also be separated into two main portions, the anomeric carbons region (δ 98–102 ppm) and the nonanomeric carbons region (δ 60–81 ppm).

#### LC–ESI–MS (liquid chromatography electrospray ionization tandem mass spectrometric)

**Figure 5:**
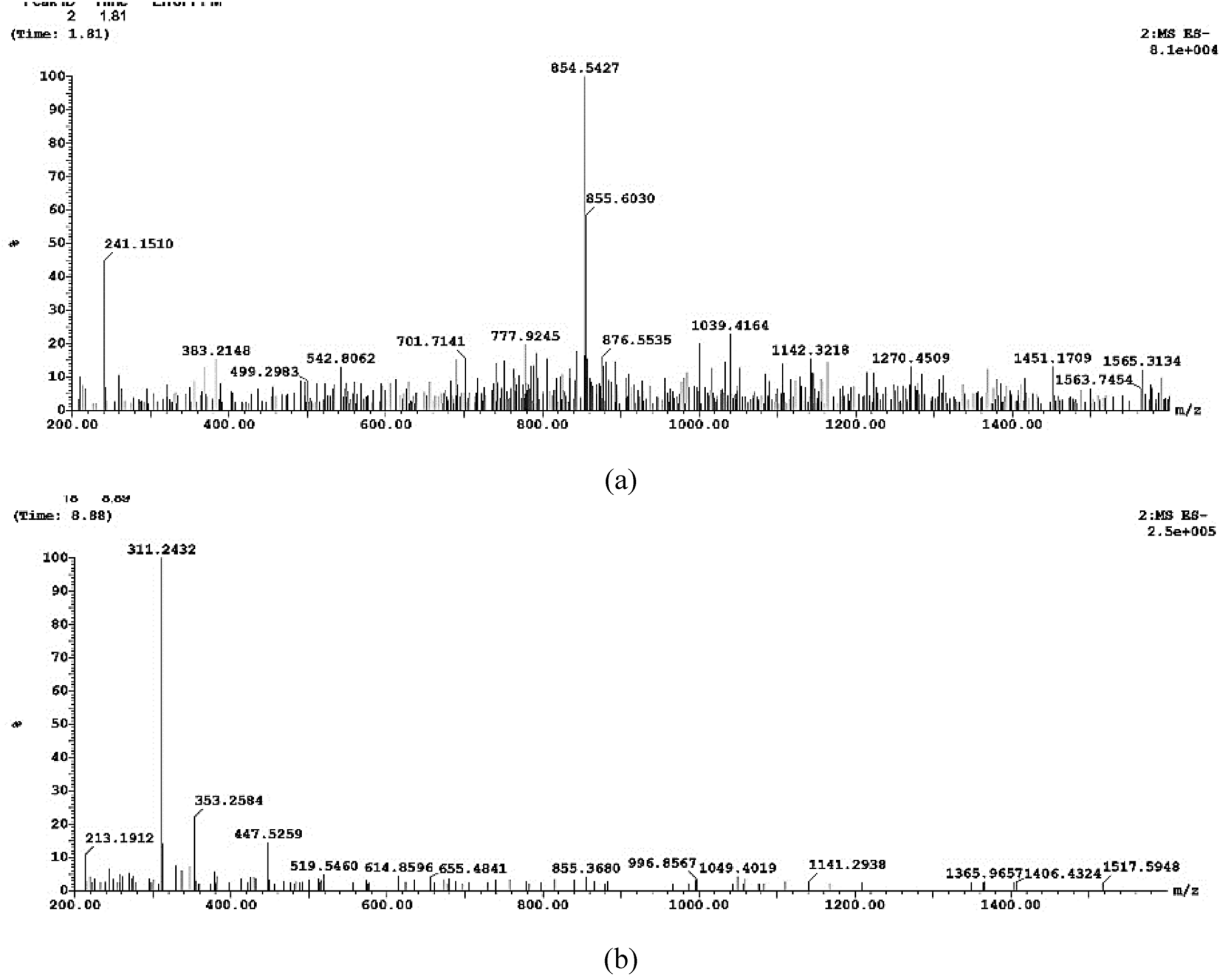
LC–ESI–MS analysis of *Lactiplantibacillus plantarum* OP218384 EPS.

LC–ESI–MS was used to identify the monomeric sugars that composed the EPS. Both positive and negative modes were performed. In the present study as shown in Fig. 5. The prominent ions at m/z 855 demonstrated glycosidic cleavage production. The ions at 655, 519, 353, and 213 m/z ascribed to hexose monosaccharide sodium adduct ions in accordance with Nunes et al.^51^. Moreover, Tudella et al.^52^ also reported the glycosidic bond cleavage for hexose oligosaccharides typical of (1–4)-linked pyranosyl units.

#### Transmission electron microscopy (TEM)

**Figure 6:**
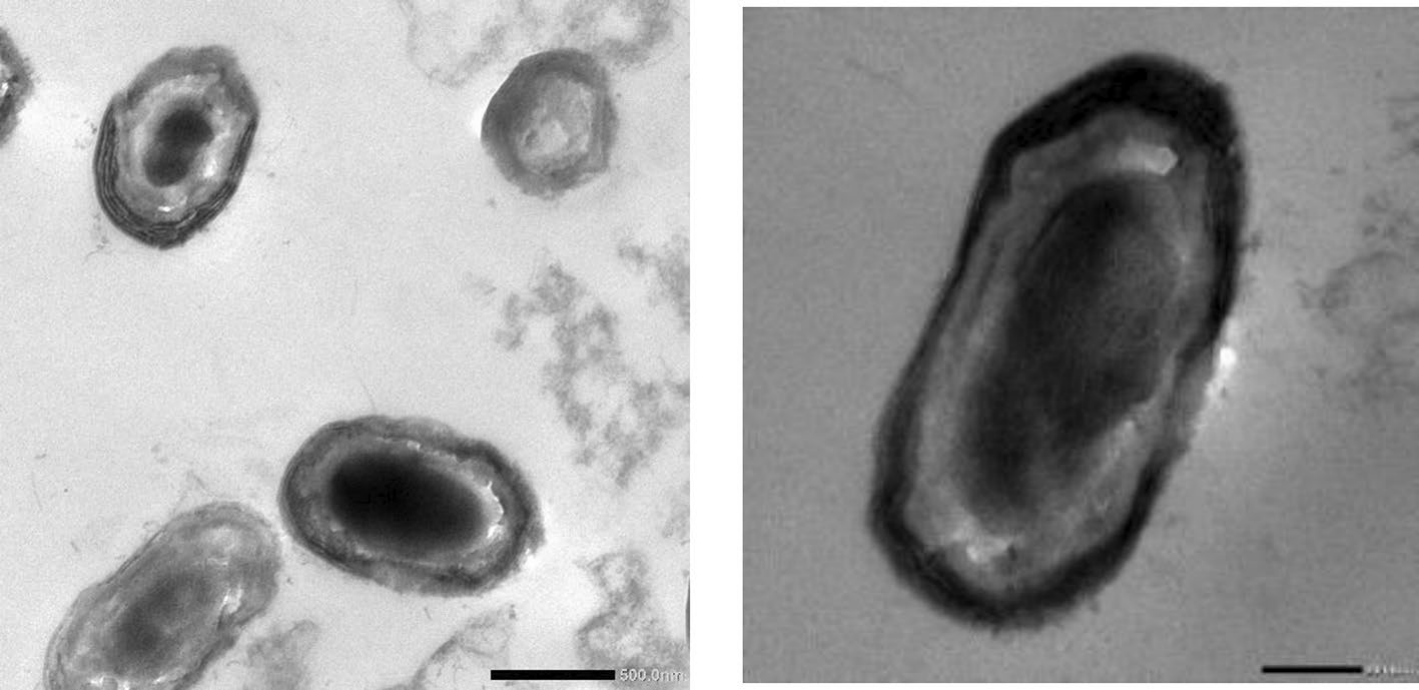
Transmission electron microscopy of *Lactiplantibacillus plantarum* OP218384 cells.

Morphological study of *Lactiplantibacillus plantarum* OP218384 cells using transmission electron microscope studies revealed the presence of capsulated cells surrounded by a regular network-like structure defined as the EPS layer (Fig. 6). There has been speculation on the potential role of EPS production as a strategy used by *L. plantarum* to adapt to certain conditions inside the gastrointestinal environment^53^.

### Optimization of biomass and EPS production using Taguchi orthogonal array

**Figure 7:**
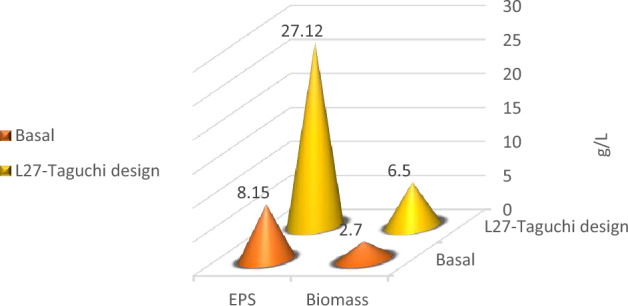
The 3D graph of the basal and L-27 Taguchi design trials for biomass and EPS production.

**Figure 8:**
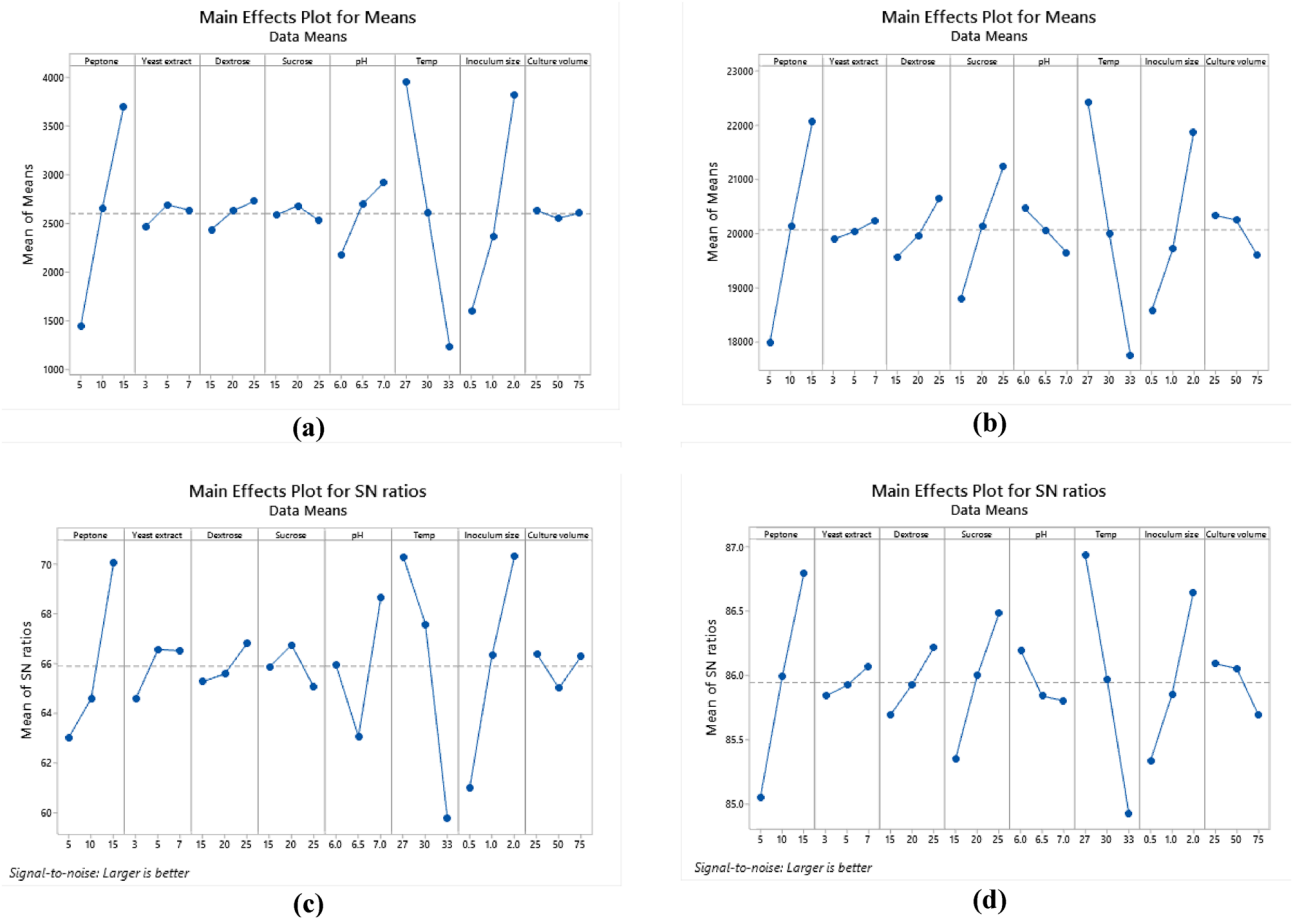
Response graph for relative factors means of biomass. (**a**), EPS (**b**). Response graph for S/N ratios for relative factors of biomass (**c**). and EPS (**d**).

Optimization of nutritional and environmental factors that lead to optimum *Lactiplantibacillus plantarum* OP218384 biomass and EPS production was carried out using Taguchi orthogonal array with 8 factors design of experiment (DOE) (Table 1). Eight variables were used namely: peptone, yeast extract, dextrose, sucrose, pH, temperature, inoculum size, and culture volume. A 27-run model was performed, the biomass and EPS response values were recorded using Mintab19 software. Statistical significance was done using the analysis of variance (ANOVA). *Lactiplantibacillus plantarum* OP218384 biomass and EPS production were significant, as indicated by F-value of the model. Trial number 23 showed the highest yield (6.5 and 27.12 g/L for biomass and EPS productions, respectively) (Table 5, Table S3) with 2.4 and 3.3 folds increase respectively (Fig. 7). The optimum level for each variable was shown in Figs. 8, (Tables S4 and S5).

**Table 5:** *Lactiplantibacillus plantarum* OP218384 biomass and EPS production using Taguchi orthogonal design.

Trials	Independent variables								Response	
	Peptone	Yeast extract	Dextrose	Sucrose	pH	Temp	Inoculum size	Culture volume	Biomass (g/L)	EPS (g/L)
1	5.0	3.0	15.0	15.0	6.0	27.0	0.5	25.0	1.03	17.56
2	5.0	3.0	15.0	15.0	6.5	30.0	1.0	50.0	1.12	16.24
3	5.0	3.0	15.0	15.0	7.0	33.0	2.0	75.0	1.26	14.42
4	5.0	5.0	20.0	20.0	6.0	27.0	0.5	50.0	1.56	19.44
5	5.0	5.0	20.0	20.0	6.5	30.0	1.0	75.0	1.38	16.89
6	5.0	5.0	20.0	20.0	7.0	33	2.0	25.0	2.01	17.53
7	5.0	7.0	25.0	25.0	6.0	27.0	0.5	75.0	1.52	20.85
8	5.0	7.0	25.0	25.0	6.5	30.0	1.0	25.0	1.46	19.65
9	5.0	7.0	25.0	25.0	7.0	33.0	2.0	50.0	1.64	19.31
10	10.0	3.0	20.0	25.0	6.0	30.0	2.0	25.0	3.25	23.45
11	10.0	3.0	20.0	25.0	6.5	33.0	0.5	50.0	0.16	17.70
12	10.0	3.0	20.0	25.0	7.0	27.0	1.0	75.0	4.04	22.03
13	10.0	5.0	25.0	15.0	6.0	30.0	2.0	50.0	3.76	22.14
14	10.0	5.0	25.0	15.0	6.5	33.0	0.5	75.0	0.52	14.87
15	10.0	5.0	25.0	15.0	7.0	27.0	1.0	25.0	4.32	21.36
16	10.0	7.0	15.0	20.0	6.0	30.0	2.0	75.0	3.39	21.36
17	10.0	7.0	15.0	20.0	6.5	33.0	0.5	25.0	0.49	16.52
18	10.0	7.0	15.0	20.0	7.0	27.0	1.0	50.0	3.96	21.87
19	15.0	3.0	25.0	20.0	6.0	33.0	1.0	25.0	1.97	20.87
20	15.0	3.0	25.0	20.0	6.5	27.0	2.0	50.0	6.32	26.36
21	15.0	3.0	25.0	20.0	7.0	30.0	0.5	75.0	3.09	20.52
22	15.0	5.0	15.0	25.0	6.0	33.0	1.0	50.0	1.32	20.23
23	15.0	5.0	15.0	25.0	6.5	27.0	2.0	75.0	6.51	27.12
24	15.0	5.0	15.0	25.0	7.0	30.0	0.5	25.0	2.88	20.86
25	15.0	7.0	20.0	15.0	6.0	33.0	1.0	75.0	1.79	18.36
26	15.0	7.0	20.0	15.0	6.5	27.0	2.0	25.0	6.33	25.33
27	15.0	7.0	20.0	15.0	7.0	30.0	0.5	50.0	3.16	19.01

*NA* not available. *S/N ratio* signal-to-noise ratio, the ratio of target value to the deviation from its mean.

**Figure 9:**
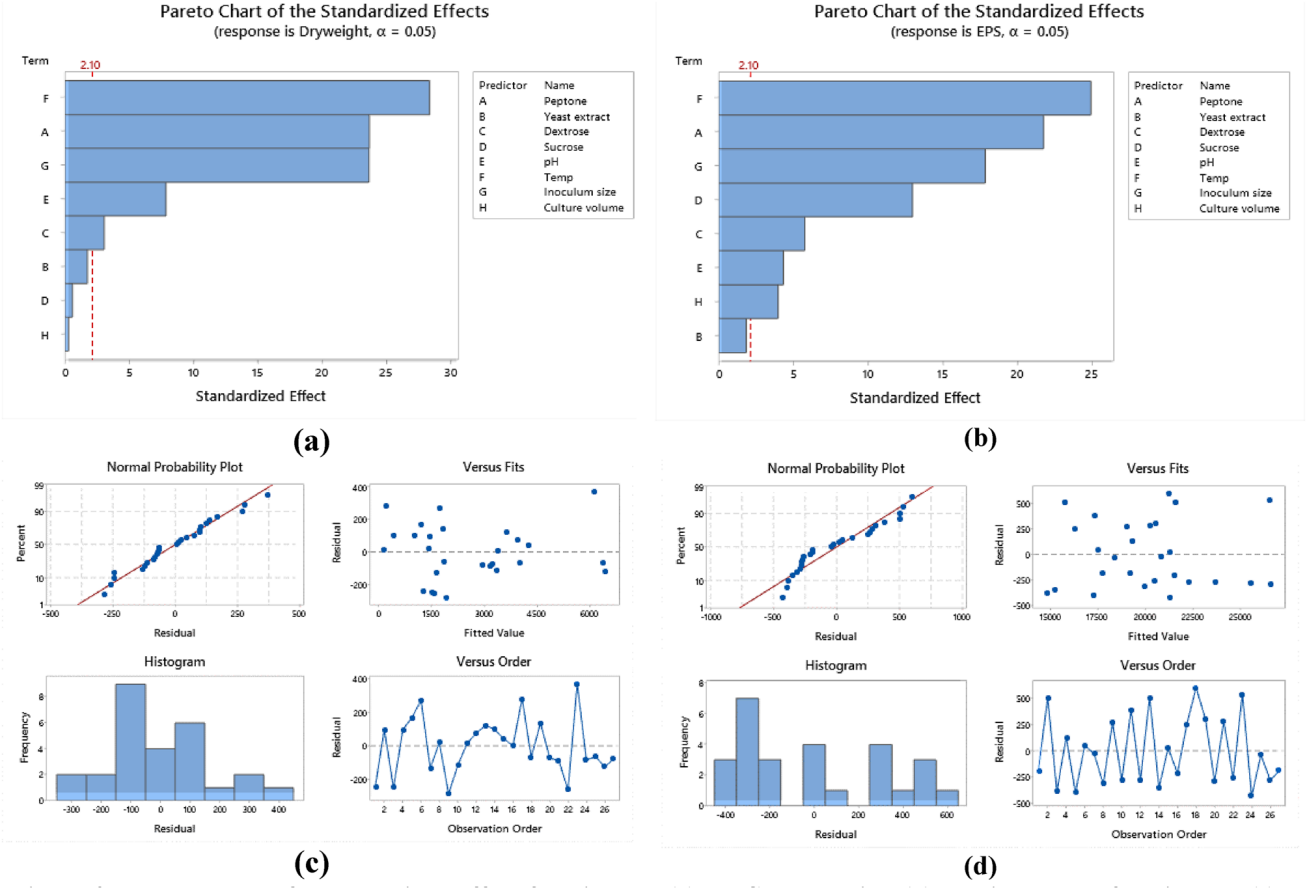
Pareto chart of standardized effect for biomass (**a**), EPS production (**b**), Residual Plot for biomass (**c),** EPS (**d**).

**Figure 10:**
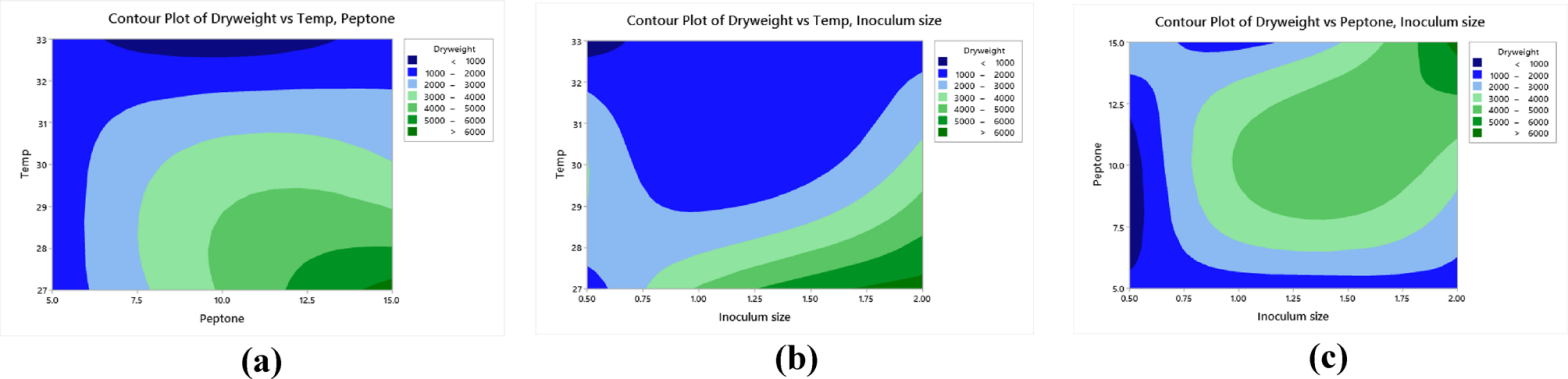
Contour plots of the relationship between biomass versus temperature and peptone (**a**), biomass versus temperature and inoculum size biomass (**b**) biomass versus peptone and inoculum size (**c**).

**Figure 11:**
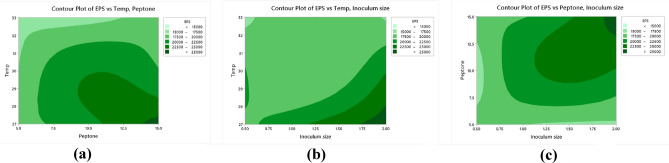
Contour plots of the relationship between: EPS versus temperature and peptone (**a**), EPS versus temperature and inoculum size (**b**), EPS versus peptone and inoculum size (**c**).

Analysis of variance (ANOVA) was used to analyze the variables interactions and the factors variation contribution of Taguchi design results. It was concluded that data in equations R1 & R2 were consistent with the experiment. Temperature showed the highest influence on the production of biomass and EPS (Fig. 9a,b) followed by peptone, inoculum size (*P* < 0.05) (Table 6, Table S6). The residual diagrams (Fig. 9c,d) indicated that the errors were regularly distributed. The studied interactions between the main effective factors were proved by the Contour plot diagrams (Figs. 10, 11). The selected factors interacted with each other to maximize the production of both biomass and EPS. The statistical experimental studies concluded that the optimum parameters that lead to maximum EPS and biomass yield were peptone, 15.0 g/L: yeast extract. 5.0 g/L; dextrose,15.0 g/L; sucrose 25.0 g/L; pH, 6.5; temperature, 27 °C; inoculum size, 2.0 and culture volume, 5.0 mL. Studies have shown that the optimal deproteinization range of LAB fermentation between 30 and 40 °C^54^. Relevant studies have shown that while an increase in temperature can enhance bacterial activity, excessively high temperatures can cause irreversible denaturation of proteins and nucleic acids in microorganisms, negatively affecting strain activity^55^.

**Table 6:** Analysis of variance for S/N ratios in relation to biomass and EPS production.

Source	Biomass production						EPS production					
	DF	Seq SS	Adj MS	F	P	% Contribution	DF	Seq SS	Adj MS	F	P	% Contribution
Peptone	2	246.50	123.248	34.56	0.000	17.382	2	13.7271	6.86606	132.48	0.000	27.764
Yeast extract	2	22.72	11.358	3.18	0.085	1.602	2	0.2480	0.12399	2.39	0.142	0.501
Dextrose	2	12.14	6.071	1.71	0.231	0.856	2	1.2610	0.63049	12.17	0.002	2.550
Sucrose	2	12.90	6.448	1.81	0.213	0.909	2	5.8288	2.91441	56.23	0.000	11.789
pH	2	141.91	70.957	19.90	0.000	10.00	2	0.8326	0.41628	8.03	0.008	1.684
Temp	2	539.78	269.890	75.65	0.000	38.064	2	18.2679	9.13396	176.24	0.000	36.949
Inoculum size	2	395.83	197.914	55.45	0.000	27.913	2	7.8583	3.92913	75.81	0.000	15.894
Culture volume	2	10.59	5.295	1.49	0.272	0.746	2	0.8936	0.44682	8.62	0.007	1.807
Residual error	10	35.71	3.571			2.518	10	0.5183	0.05183			1.048
Total	26	1418.07				100	26	49.4405				100

*DF* the total degrees of freedom, *Seq SS* sequential sums of squares, *Adj MS* adjusted sums of squares, *F* F-value. *P* p-value.

Similarly Prete et al.^56^ reported that physicochemical parameters (pH 6.5, temperature, inoculum size, culture volume and organic nitrogen sources (yeast extract, peptone)) significantly affected EPS production from LAB isolates. The primary objective of another research study was to optimize the fermentation conditions using the Plackett–Burman (PB) and response surface methodology (RSM) to enhance the EPS production^57^. Afreen et al.^57^ reported that *Lactiplantibacillus paraplantarum* NCCP 962 was identified and PB design successfully identified four independent components, including lactose, yeast extract, CaCl_2_, and tryptone, which were shown to have a statistically significant effect. The highest yield of EPS (0.910 g/L) was achieved when the lactose concentration was 6.57%, yeast extract concentration was 0.047%, CaCl_2_ concentration was 0.59%, and tryptone concentration was 1.37%. Zhang et al.^58^ study, the production process of EPS was optimized through applying initial pH 6, inoculation amount 5% (v/v), temperature 37 °C, cultivation time 36 h, glucose 3% (w/v), soy peptone 1.5% (w/v), KH_2_PO_4_ 0.3% (w/v) to reach EPS yield 0.630 mg/mL, which was 1.31 times higher than the basal conditions.

### Regression analysis modeling for biomass and EPS

Linear regression analysis in Minitab 19 software was used to develop the predictive mathematical models for the dependent variables (Eqs. 5, 6). The R^2^ value above 97% explains the higher variability and depicts the model’s validity.

5 $$ R1\;(biomass\;production) = 6.635 + 0.22656{\text{X}}1 + 0.0417{\text{X}}2 + 0.02933{\text{X}}3 - 0.00567{\text{X}}4 + 0.7522{\text{X}}5 - 0.4524{\text{X}}6 + 14817{\text{X}}7 - 0.00053{\text{X}}8 $$

**(R** ^**2**^ **= 99.11%)**

6 $$ R2\;(EPS) = 35.40 + 0.4086{\text{X}}1 + 0.0864{\text{X}}2 + 0.1083{\text{X}}3 + 0.2434{\text{X}}4 - 0.817{\text{X}}5 - 0.7798{\text{X}}6 + 2.195{\text{X}}7 - 0.01491{\text{X}}8 $$

**(R** ^**2**^ **= 98.92%)**

### Selenium nanoparticles (EPS-SeNPs) synthesis and characterization

**Figure 12:**
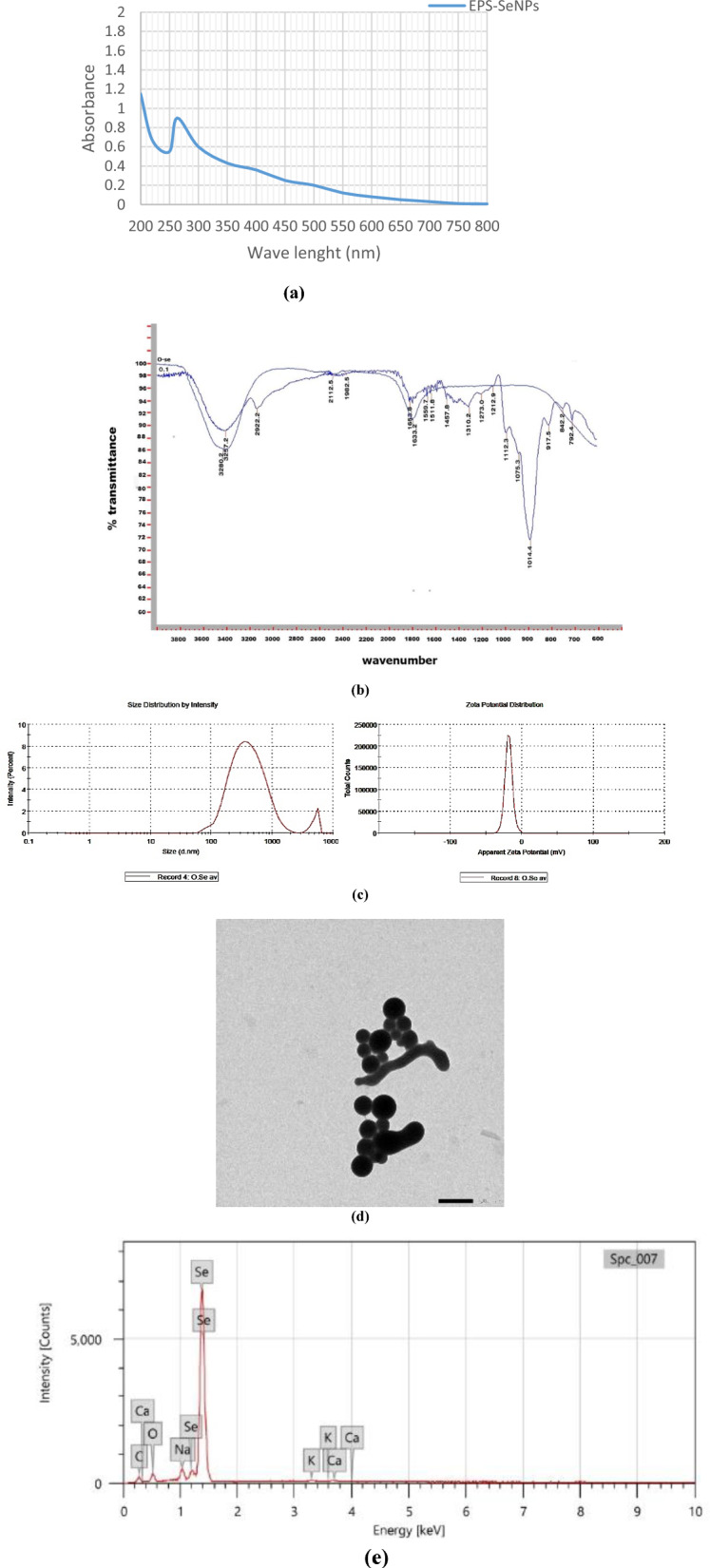
EPS-SeNPs physicochemical characterization where FTIR (**a**), UV spectrum (**b**), zeta potential, particle size (ps) and polydispersity index Zeta potential (**c**), TEM analysis (**d**) and EDX (**e**).

The optimized EPS was used as a reducing and capping agent for selenium nanoparticles (SeNPs) synthesis. The color change of the exopolysaccharide/sodium selenite (Na_2_SeO_3_) solution from colorless to orange after ascorbic acid addition proved the formation of EPS-SeNPs as described by Tang et al.^34^ (Fig. S2). The synthesized selenium nanoparticle was scanned using U.V spectroscopy to assess the characteristic peak of SeNPs at 263 nm (Fig. 12a). Tang et al.^34^ synthesized selenium nanoparticles with *Gracilaria lemaneiformis* exopolysaccharides and reported the presence of SeNPs characteristic peak around 263 nm.

FTIR analysis proved the EPS role as a coating and stabilizing agent during the synthesis of the SeNPs, through the formation of strong hydrogen bond (Se-OH). The similarity between the FTIR spectra of EPS-SeNPs and EPS indicates slight shift of the hydroxyl group position (shifted from 3257 cm^−1^ (EPS) to 3289.2 cm^−1^ (EPS-SeNPs)) (Fig. 12b). In a trial to determine the homogeneity and stability of EPS-SeNPs, zeta potential and PDI were measured (− 19.7 mv and 0.446 respectively) (Fig. 12c). The observed negative zeta potential value confirmed the EPS involvement in the formation, stabilization, and growth of well-dispersible SeNPs^34^. Moreover, the structure, size, and shape of the biosynthesized EPS-SeNPs were examined by TEM, which revealed that EPS-SeNPs had a spherical shape with size ranged from 45 to 65 nm (Fig. 12d). Similarly, Cai et al.^59^ synthesized SeNPs using *Lignosus rhinocerotis* exopolysaccharide and reported that SeNPs diameter was 50 nm.

EDX analysis of the prepared EPS-SeNPs revealed that Se percentage was 32.71%, while oxygen and carbon atoms were 14.72, 48.43% respectively (Fig. 12e). Likewise, Cao et al.^60^ synthesized selenium nanoparticles from *Grateloupia Livida* exopolysaccharides with Se atom content accounted 36.49% of the total elements.

### Antimicrobial activity of EPS-SeNPs

**Figure 13:**
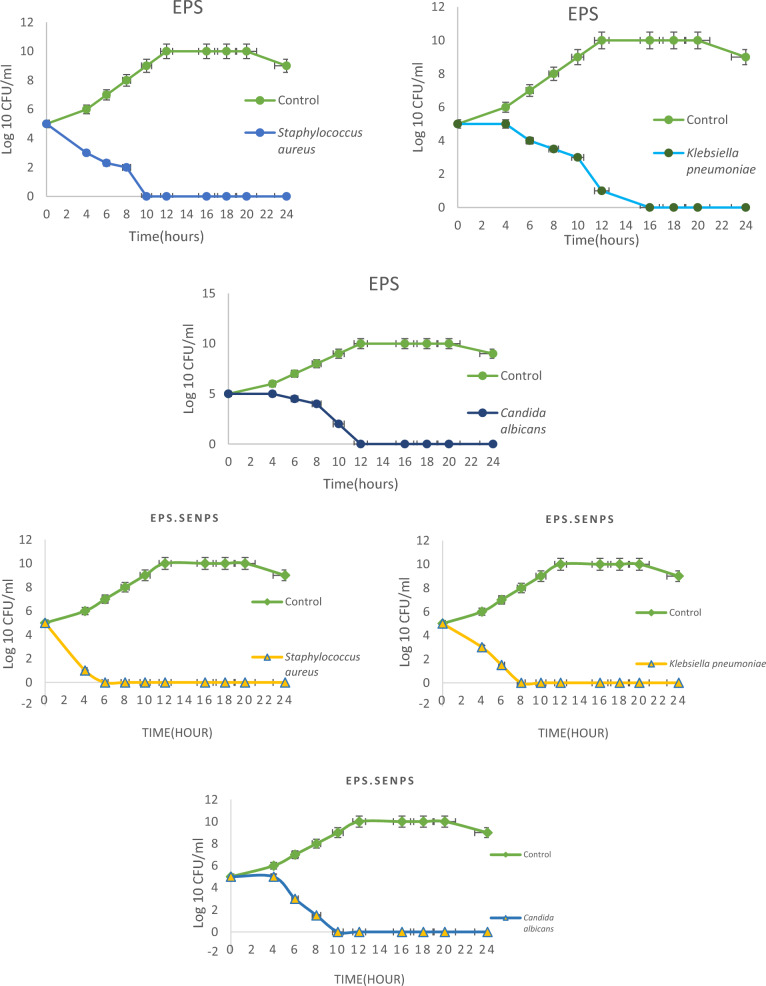
Time kill curve of the tested pathogens treated with EPS and EPS-SeNPs.

The highest antibacterial effect of EPS-SeNPs was shown against *Staphylococcus aureus* with inhibition zone 40.3 ± 0.57 mm followed by *Klebsiella pneumoniae,* MRSA *and E. coli* (35.3, 35 and 30.7 mm respectively) (Fig. S3). The prepared nanoparticles showed strong antifungal effect against *Candida albicans* (49.6 ± 0.57 mm) (Table 7). MIC index values of the synthesized EPS-SeNPs revealed a bactericidal and fungicidal effect against the tested pathogens. Microbial time kill curve study revealed the full eradication of microbial growth after 6, 8 and 10 h against *Staphylococcus aureus*, *E. coli*, *Candida albicans* and *Klebsiella pneumoniae* respectively (Fig. 13).

**Table 7:** Antimicrobial activity, MIC, MBC, and MIC index of the tested EPS and EPS-SeNPs.

Tested pathogen	EPS				Na_2_SeO_3_				EPS-SeNPs			
	IZ (mm)	MIC (μg/mL)	MBC (μg/mL)	MIC index	IZ (mm)	MIC (μg/mL)	MBC (μg/mL)	MIC index	IZ (mm)	MIC (μg/mL)	MBC (μg/mL)	MIC index
*E. coli*	5.5	32	128	4	13.0	32	128	4	30.7	16	64	4
*Ent. aerogenes*	8.0	64	256	4	19.0	16	64	4	33.0	4	16	4
*Staphylococcus aureus*	8.0	32	128	4	17.0	16	64	4	40.3	4	32	8
*Klebsiella pneumoniae*	7.0	32	128	4	15.0	32	128	4	35.3	4	32	8
MRSA	8.0	16	64	4	R	256	256	1	35.0	8	32	4
*Candida albicans*	8.0	32	128	4	18.0	16	128	8	49.6	8	32	4

*MIC* minimum inhibitory concentration, *MBC* minimum bactericidal concentration.

El-Deeb et al.^61^ reported that SeNPs exhibited antimicrobial effect against *Staphylococcus aureus*, MRSA and *E. coli* with inhibition zone diameter 29, 27 and 13 mm respectively. Moreover, Iqbal et al.^38^ declared that SeNPs showed antibacterial activity against *Klebsiella pneumoniae*, *E. coli* and *Staphylococcus aureus* with inhibition zone diameters 24, 23 and 22 mm respectively. According to Hashem and Salem^37^ SeNPs antimicrobial mechanism of action has four pathways: (1) metabolic invasion through disruption of intracellular adenosine triphosphate (ATP) levels, (2) fluctuation of intracellular reactive oxygen species (ROS) values, (3) depolarization, and (4) disruption of biological membranes.

### Antioxidants, and cytotoxicity effects of EPS and EPS-SeNPs

#### Antioxidant properties

EPS and EPS-SeNPs showed concentration-dependent DPPH free radical scavenging effect at different concentrations ranged from 2.5 to 100 µg/mL (Table 8). The obtained data revealed that the highest antioxidant capacity was detected with Trolox (positive control) followed by EPS-SeNPs and EPS respectively. On the other hand, at 100 µg/mL, the DPPH free radical scavenging ability of the EPS-SeNPs reached 97.4% exceeding the EPS and the positive control percentage (93.1 and 92.9%, respectively) which verified the higher activity of the synthesized nanoparticles. The noticed EPS scavenging activity could be attributed to the presence of hydroxyl group, which can donate electrons to decrease the radicals concentration reaching a more stable state^17^. In accordance with Gunti et al.^62^ reported that the antioxidant activity of Phyto fabricated selenium nanoparticles (PF-SeNPs) was dependent on surface functional molecules and the size of nanoparticles occupied by secondary metabolites. PF-SeNPs also assisted in the protection of cells from free radicals by activating some selenoenzymes such as glutathione peroxidase.

**Table 8:** DPPH radical scavenging activity of EPS, EPS-SeNPs and Trolox.

Conc (µg/mL)	EPS	EPS-SeNPs	Trolox (positive control)
100	93.1 ± 0.01^e^	97.4 ± 0.02^e^	92.9 ± 0.01^e^
50	73 ± 0.02^d^	76.2 ± 0.05^d^	84.1 ± 0.04^d^
10	39.8 ± 0.02^c^	57.1 ± 0.03^c^	62.8 ± 0.02^c^
5	14.3 ± 0.01^b^	18.9 ± 0.07^b^	27.7 ± 0.01^b^
2.5	0.5 ± 0.001^a^	1.2 ± 0.003^a^	0.9 ± 0.002^a^
EC_50_	182.8 ± 10	133.7 ± 7.5	82.41 ± 4.6

Different letters a, b and c within the same column indicate that they are significantly different at p < 0.05 (letter a is the smallest, followed by b, c, d and finally the letter e is the highest one).

#### Cytotoxic properties

**Figure 14:**
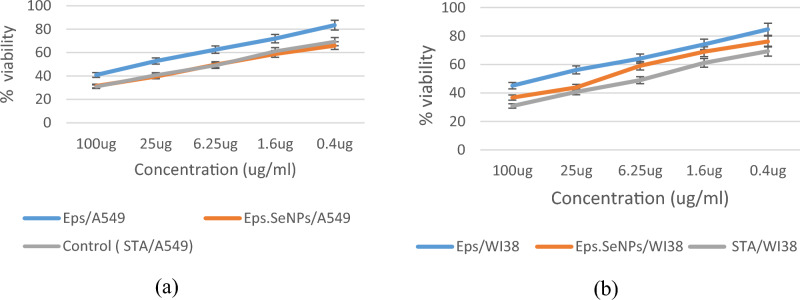
Effect of EPS, EPS-SeNPs and Staurosporine levels against Cell viability of A549 (**a**) and WI38 (**b**) respectively.

To assess the safety of EPS and EPS-SeNPs, in vitro cytotoxicity against WI38 cells was investigated and compared to that of Staurosporine. In order to investigate the anti-lung cancer properties, cytotoxic IC_50_ was calculated against A549 cells. The effect of EPS and EPS-SeNPs against A549 and WI38 cell viability showed a dose-dependent decrease with the concentration ranging from 0.4 to 100 μg/mL (Fig. 14). The IC_50_ of EPS and EPS-SeNPs against A549 were 32.06 ± 1.57 and 5.324 ± 0.26 µg/mL, respectively, while the IC_50_ toward WI38 were 52.03 ± 2.29 and 15.83 ± 0.7 µg/mL, respectively (Table 9) which was in line with Tang et al.^34^ results. However, Staurosporine showed the highest therapeutic index with higher anti-lung cancer effect when compared to EPS-SeNPs. The efficacy of the synthesized EPS-SeNPs against cancer cells, A549, was higher than that of crude EPS, establishing the way for its usage as a therapeutic agent against lung carcinoma. The estimated therapeutic index showed that EPS-SeNPs were significantly safer and more effective against A549 cells than crude EPS. Tang et al.^63^ stated that arabinogalactans/selenium nanoparticles (LAG-SeNPs) showed the highest cytotoxic effect against A549 cells, followed by HepG-2 and MCF-7 cells. It was revealed that anti-cancer efficiency of SeNPs was improved via decoration with EPS, which improved the cellular absorption and permeability of selenium nanoparticles. Also, it's worth mentioning that their efficiency was inversely correlated with their size^4,10^.

**Table 9:** The IC50 and the therapeutic index of EPS, EPS-SeNPs and Staurosporine against A549, WI38.

Sample	Cytotoxicity, IC_50_ (µg/mL)		Therapeutic index
	A549	WI38	WI38/A549
EPS	32.06 ± 1.57^c^	52.03 ± 2.29^c^	1.62 ± 0.11^a^
EPS-SeNPs	5.324 ± 0.26^a^	15.83 ± 0.7^a^	2.97 ± 0.21^b^
Staurosporine	6.435 ± 0.31^b^	27.17 ± 1.19^b^	4.22 ± 0.23^c^

Different letters a, b and c within the same column indicate that they are significantly different at p < 0.05 (letter a is the smallest, followed by b and finally the letter c is the highest one).

#### Effect of EPS and EPS-SeNPs on cellular reactive oxygen species (ROS)

Nowadays, many anticancer medicines present on the market cause tumor cell death by raising ROS levels. The green synthesis of SeNPs that induced the ROS levels in cancer cells seems to be promising therapy^41^. Results showed that EPS-SeNPs were more effective compared with EPS and control, Table 10 showed the ∆RFU, control% and relative fold-increase in ROS levels against A549 which reached 1.26, 1.13 and 1 respectively. It was reported that the overproduction of ROS has been linked to mitochondrial malfunction, including mitochondrial membrane potential (MMP) loss and activation of the mitochondrial apoptosis pathway^41,62,64^.

**Table 10:** ROS generation in A549 cell line exposed to EPS and EPS-SeNPs.

Sample	ROS		
	∆RFU	control %	Fold increase
EPS/A549	162,119	113.582	1.13
EPS-SeNPs/A549	179,851	126.005	1.26
H_2_O_2_/A549	218,125	152.82	1.52
Cont.A549	142,733	100	1

*∆RFU* relative fluorescence unit.

#### Real time RT–PCR

##### The relative change in the genetic expression of proapoptotic (Caspase-3 and BAX) and anti-apoptotic (Bcl2) genes

According to Nakamura and Takada^64^ excess ROS generation can damage the cellular proteins, nucleic acids, lipids, membranes, and organelles, which stimulates cell death processes, including apoptosis. Excessive mitochondrial ROS can cause intrinsic apoptosis, which activates caspase 9 expression. Caspase 9, a critical component in the intrinsic route, subsequently activates caspase 3, 6, and 7, resulting in cellular protein breakage and apoptosis. The mentioned pathways lead to inhibiting the anti-apoptotic activity of Bcl2's and activate Bax. RT-PCR data (Table 11) revealed the proapoptotic potency of EPS and EPS-SeNPs for caspase 3 and Bax expressions. Caspase 3 expression reached 3.75 and 6.34 fold in EPS and EPS-SeNPs treated A549 cells, respectively. Furthermore, Bax expression was increased by 3.258 and 4.969 fold in EPS and EPS-SeNPs treated A549 cells, respectively, while Bcl2's anti-apoptotic gene was decreased by 0.636 and 0.324 fold, respectively.

**Table 11:** Effect of EPS and EPS-SeNPs on expression of proapoptotic (Casp3 and Bax) and anti-apoptotic (Bcl-2) genes using RT-PCR.

Sample	Fold change		
	Casp3	Bax	Bcl2
EPS/A549	3.75 ± 0.03^b^	3.26 ± 0.01^b^	0.64 ± 0.13^b^
EPS-SeNPs/A549	6.34 ± 0.08^c^	4.97 ± 0.03^c^	0.32 ± 0.09^a^
Cont.A549	1.00 ± 0.003^a^	1.00 ± 0.005^a^	1.00 ± 0.001^c^

Within the same column, mean with different letter a, b and c are significantly different at p < 0.05 where the mean with letter a in the smallest one followed by b and finally the mean with letter c is the highest one.

#### Cell cycle analysis assay

**Figure 15:**
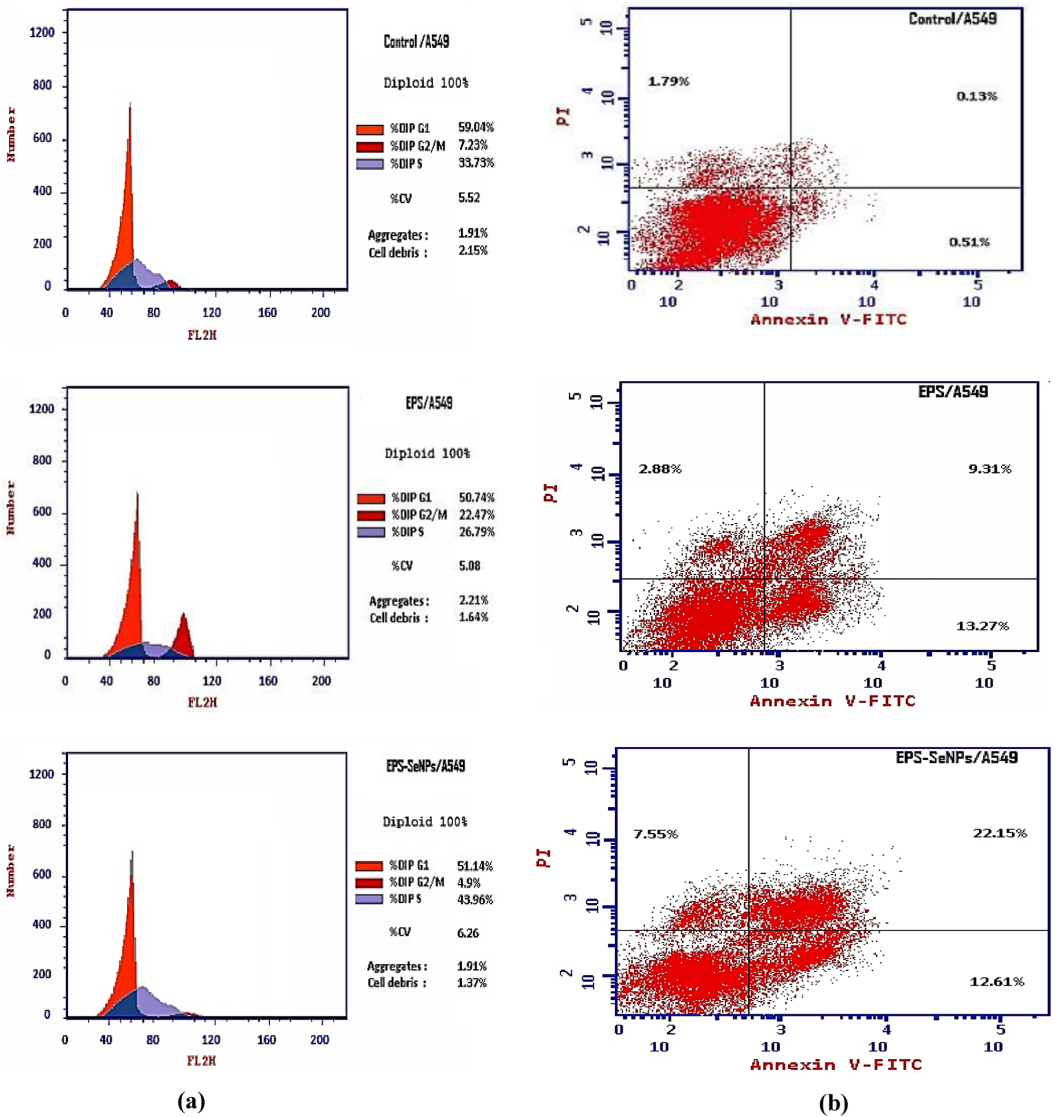
Flow cytometric profile of A549 cells for control, treated with EPS and EPS-SeNPs (**a**), Annexin V-FITC/PI double staining technique for quantitative study of apoptotic cells cytometry of A549 cells control, treated with EPS and EPS-SeNPs (**b**).

Apoptosis has been considered one of the most important mechanisms for anticancer activity^65^. Apoptosis is a process of planned cell death in multicellular organisms. Unlike necrosis, which is a kind of violent cell death triggered by acute cellular damage, apoptosis is a tightly regulated and controlled process that benefits the organism. Most cancer therapies essentially induce apoptosis in targeted cancer cells to cause cancer cells death^63^. SeNPs come in various forms, and each form has a unique anticancer mechanism. Therefore, flow cytometry was employed to investigate the anticancer mechanism of EPS and EPS-SeNPs against A549 cells. The results showed that apoptosis of A549 cells was remarkably induced after treatment with EPS and EPS-SeNPs. Compared with the control group, EPS-SeNPs showed higher total apoptosis and necrosis (42.31 and 7.55 respectively), than EPS (25.46 and 2.88 respectively) (Table 12 and Fig. 15). Inhibition of cell proliferation activity is associated with the blocking of the cell cycle. The A549 cell cycle distribution was investigated with EPS and EPS-SeNPs to determine whether cell cycle arrest was related to EPS and EPS-SeNPs proliferation inhibitory effect. Compared with the control group, EPS caused cell growth arrest at the G2/M phase, while EPS-SeNPs caused cell growth arrest in the S phase (Table 13). Jolly et al.,^66^ explained that SeNPs' anticancer properties are attributed to selenium's activation of glutathione S-transferase (GST) hence inhibiting cancer cell development by causing cell cycle arrest at the S phase. Through the process of endocytosis, cancer cells specifically take up SeNPs, that cause activating the signal transduction pathways associated with apoptosis, causing cancer cells death. Wu et al.^67^ indicated that A549 cells' proliferation was considerably decreased by *Polyporus rhinocerus* water-soluble polysaccharides-protein complexes selenium nanoparticles (PRW-SeNPs) through the activation of apoptosis and G2/M phase arrest, DNA fragmentation, nuclear condensation and chromatin condensation were seen during the TUNEL-DAPI co-staining experiment, which further supported the activation of apoptosis. Ferro et al.^68^ demonstrated that SeNPs stabilized with 1,6-D-glucan have also shown anticancer activities against HeLa cells (a cervical cancer cell line), triggering apoptosis through the mitochondrial intrinsic route, decreasing the mitochondrial membrane potential and arresting cell division during the S phase.

**Table 12:** Apoptosis and necrosis in A549 cells treated with EPS and EPS-SeNPs.

	Sample	Apoptosis %			Necrosis%
		Total	Early	Late	
1	EPS/A549	25.46	13.27	9.31	2.88
2	EPS-SeNPs/A549	42.31	12.61	22.15	7.55
3	Cont.A549	2.43	0.51	0.13	1.79

**Table 13:** Cell cycle progression of A549 cells affected by EPS and EPS-SeNPs.

Sample	DNA content		
	%G0-G1	%S	%G2/M
EPS/A549	50.74	26.79	22.47
EPS-SeNPs/A549	51.14	43.96	4.90
Cont.A549	59.04	33.73	7.23

## Conclusion

Data concluded that *Lactiplantibacillus plantarum* strain A2 with accession number OP218384 was the most potent EPS producer. The protein content of *Lactiplantibacillus plantarum* OP218384 crude EPS was 3.71 ± 0.55 mg/g, while total carbohydrate content ranged from 68.963 to 76.879 mg/g. Taguchi design optimization produced 27.12 g/L of *L. plantarum* strain A2—EPS which was higher than the previously reported data. *L. plantarum* strain A2—EPS was used as a capping and stabilizing agent for SeNPs which showed significant bactericidal and fungicidal action. Finally, the synthesized nanoparticles (EPS-SeNPs) demonstrated a strong antioxidant and anticancer effect against A549 lung cell line, as well as growth inhibition and anti-apoptotic activities. Overall, this proved that the isolated strain is a super probiotic strain with potent antibacterial effect, has high EPS production yield and significant ability to synthesize and stabilize selenium nanoparticles.

### Supplementary Information

Supplementary Information.

## Data Availability

The datasets analyzed during the current study are available from the National Center for Biotechnology Information (NCBI) database. https://www.ncbi.nlm.nih.gov/nuccore/OP218384.1/ (accessed on 19 August 2022).
