# Supplementary material for: Selenium nanoparticles coated bacterial polysaccharide with potent antimicrobial and anti-lung cancer activities
Source: Sci Rep. 2023 Dec 10;13:21871. doi: 10.1038/s41598-023-48921-9 (PMC10711019; doi:10.1038/s41598-023-48921-9)
Supplement: Supplementary file 1 — Supplementary Information. [file 41598_2023_48921_MOESM1_ESM.docx]

**Supplementary file**

| 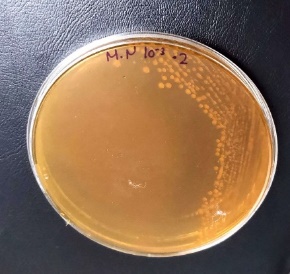 | **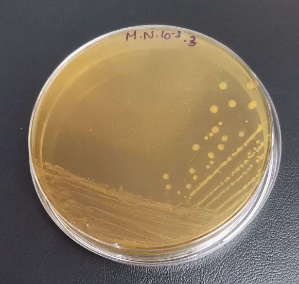** | **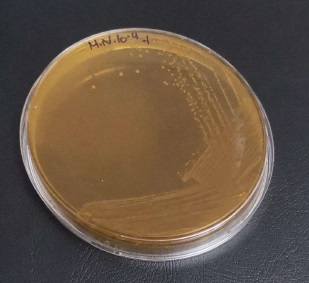** |
| --- | --- | --- |
| **(a)** | | |
| 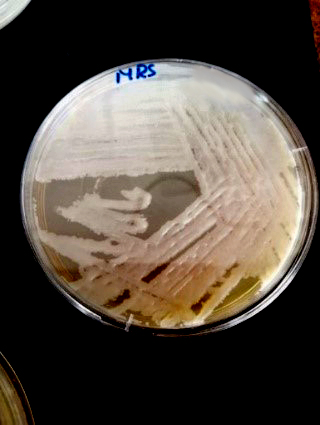 | **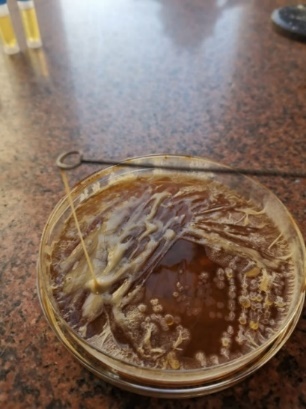** | **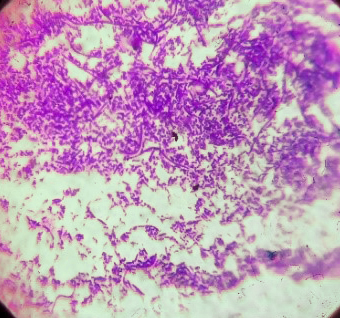** |
| **(b)** | **(c)** | **(d)** |
| Figure S1. isolates from different samples on MRS plates (a), most potent isolate no.1 (b), showing ropiness of isolate(c) gram stain for isolates under light microscope (d). | | |

| 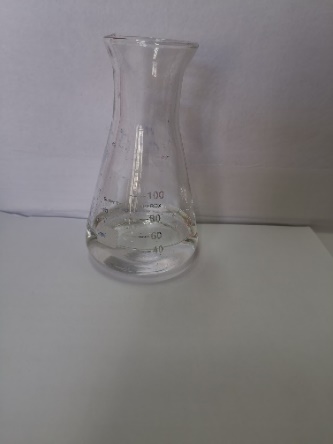  (a) | 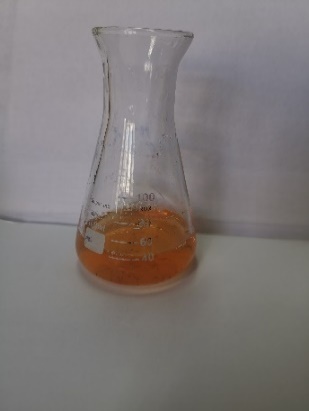  (b) | 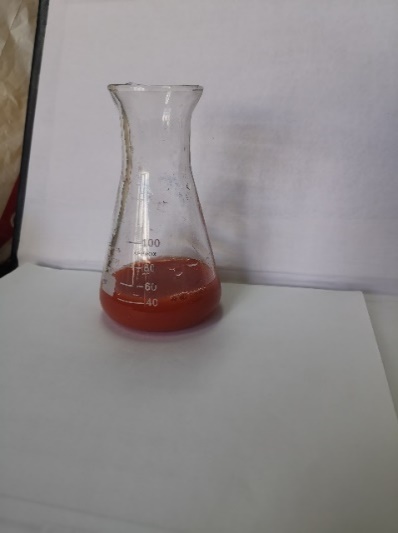  (c) |
| --- | --- | --- |
| Figure S2. green synthesis production of EPS-SeNPs the color change from colorless to dark orange | | |

| **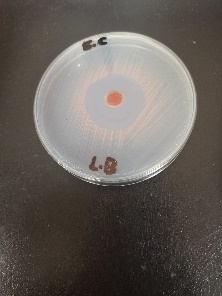** | **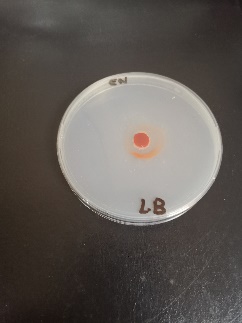** | **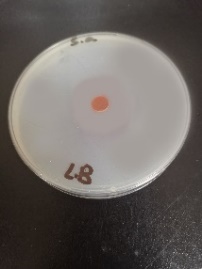** | **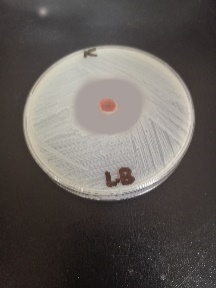** | **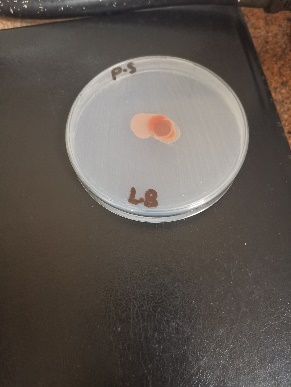** | **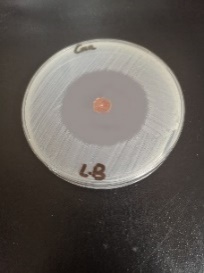** |
| --- | --- | --- | --- | --- | --- |
| ***E. coli*** | ***Ent. aerogenes*** | ***Staphylococcus aureus*** | ***Klebsiella pneumoniae*** | ***P. vulgaris*** | ***Candida albicans*** |
| Figure S3.: showing antimicrobial activity of EPS-SeNPs against *E. coli,* *Klebsiella pneumoniae*, *Staphylococcus aureus* and *Candida albicans* | | | | | |

**Table S 1: Morphological, physiological and biochemical characteristics of LAB isolates**

| No. | Source | Isolate Code | Colony morphology | Gram Stain | Cell shape | Biochemical parameter | | colony color on Congo red agar |
| --- | --- | --- | --- | --- | --- | --- | --- | --- |
|  |  |  |  |  |  | Catalase | Coagulase |  |
| 1 | **Raw buffalo milk** | Isolate no.1 | Entire, circular white to creamy color | Gram positive | Bacilli | negative | negative | black |
| 2 |  | Isolate no.2 | Entire, circular white to creamy color | Gram positive | Bacilli | negative | negative | black |
| 3 |  | Isolate no.3 | Entire, circular white to creamy color | Gram positive | Bacilli | negative | negative | black |
| 4 |  | Isolate no.4 | Entire, circular white to creamy color | Gram positive | Bacilli | negative | negative | dark red |
| 5 | **Raw caw milk** | Isolate no.5 | Entire, circular white to creamy color | Gram positive | Bacilli | negative | negative | black |
| 6 |  | Isolate no.6 | Entire, circular white to creamy color | Gram positive | Coccobacilli | negative | negative | black |
| 7 |  | Isolate no.7 | Entire, circular white to creamy color | Gram positive | Bacilli | negative | negative | black |
| 8 |  | Isolate no.8 | Entire, circular white to creamy color | Gram positive | short bacilli | negative | negative | dark red |
| 9 | **Yogurt** | Isolate no.9 | Entire, circular white to creamy color | Gram positive | short bacilli | negative | negative | dark red |
| 10 |  | Isolate no.10 | Entire, circular white to creamy color | Gram positive | Coccobacilli | negative | negative | dark red |
| Samples coded (O), buffalo milk. (N), caw milk. (N.3-N.4) ,yogurt samples . | | | | | | | | |

**Table S 2: Tolerance of lactic acid bacteria isolates to different pH and bile salt concentrations**

| **No.** | **LAB**  **Isolate Code** | **Growth at different pH** | | | **Growth at different**  **Bile salt %** | | |
| --- | --- | --- | --- | --- | --- | --- | --- |
|  |  | **2** | **3** | **4** | **0.3** | **1** | **2** |
| 1 | Isolate no.1 | **+** | **++** | **+++** | **+++** | **++** | **+** |
| 2 | Isolate no.2 | **+** | **++** | **++** | **++** | **++** | **+** |
| 3 | Isolate no.3 | **+** | **+** | **++** | **+** | **+** | **-** |
| 4 | Isolate no.4 | **+** | **++** | **++** | **++** | **+** | **-** |
| 5 | Isolate no.5 | **+** | **+** | **+** | **+++** | **++** | **+** |
| 6 | Isolate no.6 | **+** | **++** | **+++** | **++** | **++** | **+** |
| 7 | Isolate no.7 | **-** | **+** | **++** | **++** | **+** | **+** |
| 8 | Isolate no.8 | **+** | **++** | **++** | **++** | **+** | **-** |
| 9 | Isolate no.9 | **+** | **++** | **+++** | **++** | **-** | **-** |
| 10 | Isolate no.10 | **++** | **++** | **++** | **++** | **+** | **-** |
| **LAB, lactic acid bacteria. +++ high growth. ++, moderate growth. +, week growth. -, no growth.** | | | | | | | |

**Table S 3: Biomass, EPS response and its signal to noise ratio**

|  | **response** | | | |
| --- | --- | --- | --- | --- |
| **Trials** | **Biomass**  **(g/L)** | **EPS**  **(g/L )** | **S/N ratios of Dry weight** | **S/N ratios of EPS** |
| 1 | 1.03 | 17.56 | 0.26 | *24.89* |
| 2 | 1.12 | 16.24 | 0.98 | 24.21 |
| 3 | 1.26 | 14.42 | 2.01 | 23.18 |
| 4 | 1.56 | 19.44 | 3.86 | 25.77 |
| 5 | 1.38 | 16.89 | 2.80 | 24.55 |
| 6 | 2.01 | 17.53 | 6.06 | 24.88 |
| 7 | 1.52 | 20.85 | 3.64 | 26.38 |
| 8 | 1.46 | 19.65 | 3.29 | 25.87 |
| 9 | 1.64 | 19.31 | 4.30 | 25.72 |
| 10 | 3.25 | 23.45 | 10.24 | 27.40 |
| 11 | 0.16 | 17.70 | 15.9176- | 24.96 |
| 12 | 4.04 | 22.03 | 12.13 | 26.86 |
| 13 | 3.76 | 22.14 | 11.50 | 26.90 |
| 14 | 0.52 | 14.87 | -5.68 | 23.45 |
| 15 | 4.32 | 21.36 | 12.71 | 26.59 |
| 16 | 3.39 | 21.36 | 10.60 | 26.59 |
| 17 | 0.49 | 16.52 | 6.1961- | 24.36 |
| 18 | 3.96 | 21.87 | 11.95 | 26.80 |
| 19 | 1.97 | 20.87 | 5.89 | 26.39 |
| 20 | 6.32 | 26.36 | 16.01 | 28.42 |
| 21 | 3.09 | 20.52 | 9.80 | 26.24 |
| 22 | 1.32 | 20.23 | 2.41 | 26.12 |
| 23 | 6.51 | 27.12 | 16.27 | 28.67 |
| 24 | 2.88 | 20.86 | 9.19 | 26.39 |
| 25 | 1.79 | 18.36 | 5.06 | 25.28 |
| 26 | 6.33 | 25.33 | 16.03 | 28.07 |
| 27 | 3.16 | 19.01 | 9.99 | 25.58 |

**Table S 4. Response Table for Signal to Noise Ratios and Means for variables for biomass production (Larger is better)**

|  | **Response Table for Signal to Noise Ratios for variables** | | | | | | | | **Response Table for variables Means** | | | | | | | |
| --- | --- | --- | --- | --- | --- | --- | --- | --- | --- | --- | --- | --- | --- | --- | --- | --- |
| **Level** | **Peptone** | **Yeast extract** | **Dextrose** | **Sucrose** | **pH** | **Temp** | **Inoculum size** | **Culture volume** | **Peptone** | **Yeast extract** | **Dextrose** | **Sucrose** | **pH** | **Temp** | **Inoculum size** | **Culture volume** |
| 1 | 3.025 | 4.5599 | 5.2757 | 5.8734 | 5.9399 | 10.3179 | 0.9937 | 6.3849 | 1.442 | 2.471 | 2.440 | 25.88 | 2.177 | 3.954 | 1.601 | 2.638 |
| 2 | 4.5937 | 6.5698 | 5.5834 | 6.7543 | 3.0655 | 7.5995 | 6.3576 | 5.0115 | 2.655 | 2.696 | 2.631 | 2.685 | 2.699 | 2.610 | 2.373 | 2.556 |
| 3 | 10.0725 | 6.5179 | 6.8286 | 5.0599 | 8.6822 | -0.2297 | 10.3364 | 6.2913 | 3.707 | 2.638 | 2.733 | 2.531 | 2.928 | 1.240 | 3.830 | 2.611 |
| Delta | 7.0510 | 1.9699 | 1.5529 | 1.6944 | 5.6168 | 10.5477 | 9.3427 | 1.3734 | 2.265 | 0.224 | 0.293 | 0.154 | 0.752 | 2.714 | 2.230 | 0.82 |
| Rank | 3 | 5 | 7 | 6 | 4 | 1 | 2 | 8 | 2 | 6 | 5 | 7 | 4 | 1 | 3 | 8 |

**Table S 5. Response Table for Signal to Noise Ratios and Means for variables for EPS production (Larger is better)**

|  | **Response for Signal to Noise Ratios for variables** | | | | | | | | **Response for variables Means** | | | | | | | | |
| --- | --- | --- | --- | --- | --- | --- | --- | --- | --- | --- | --- | --- | --- | --- | --- | --- | --- |
| **Level** | **Peptone** | **Yeast extract** | **Dextrose** | **Sucrose** | **pH** | **Temp** | **Inoculum size** | **Culture volume** | | **Peptone** | **Yeast extract** | **Dextrose** | **Sucrose** | **pH** | **Temp** | **Inoculum size** | **Culture volume** |
| 1 | 25.05 | 25.84 | 25.69 | 25.35 | 26.19 | 26.94 | 25.34 | 26.09 | | 17.99 | 19.91 | 19.58 | 18.81 | 20.47 | 22.4 | 18.59 | 20.35 |
| 2 | 25.99 | 25.92 | 25.93 | 26.00 | 25.84 | 25.97 | 25.85 | 26.05 | | 20.14 | 20.05 | 19.97 | 20.15 | 20.08 | 20.01 | 19.72 | 20.26 |
| 3 | 26.79 | 26.07 | 26.22 | 26.48 | 25.80 | 24.93 | 26.65 | 25.69 | | 22.07 | 20.66 | 20.66 | 21.24 | 19.66 | 17.76 | 21.89 | 19.60 |
| Delta | 1.75 | 0.23 | 0.53 | 1.13 | 0.39 | 2.01 | 1.31 | 0.40 | | 4.09 | 0.35 | 1.08 | 2.43 | 0.82 | 4.68 | 3.30 | 0.75 |
| Rank | 2 | 8 | 5 | 4 | 7 | 1 | 3 | 6 | | 2 | 8 | 5 | 4 | 6 | 1 | 3 | 7 |

**Table S 6. Analysis of Variance regression for biomass production and EPS production**

|  | **biomass production** | | | | | **EPS production** | | | | |
| --- | --- | --- | --- | --- | --- | --- | --- | --- | --- | --- |
| **Source** | **DF** | **Adj SS** | **Adj MS** | **F-Value** | **P-Value** | **DF** | **Adj SS** | **Adj MS** | **F-Value** | **P-Value** |
| Regression | 8 | 82.389 | 10.2980 | 250.00 | 0.000 | 8 | 262.203 | 32.7754 | 205.97 | 0.000 |
| Peptone | 1 | 23.0973 | 23.0973 | 560.73 | 0.000 | 1 | 75.113 | 75.1129 | 472.03 | 0.000 |
| Yeast extract | 1 | 0.1250 | 0.1250 | 3.03 | 0.099 | 1 | 0.537 | 0.5373 | 3.38 | 0.083 |
| Dextrose | 1 | 0.3872 | 0.3872 | 9.40 | 0.007 | 1 | 5.281 | 5.2813 | 33.19 | 0.000 |
| Sucrose | 1 | 0.0145 | 0.0145 | 0.35 | 0.561 | 1 | 26.669 | 26.6693 | 167.60 | 0.000 |
| pH | 1 | 2.5463 | 2.5463 | 61.82 | 0.000 | 1 | 3.001 | 3.0012 | 18.86 | 0.000 |
| Temp | 1 | 33.1569 | 33.1569 | 804.95 | 0.000 | 1 | 98.514 | 98.5140 | 619.09 | 0.000 |
| Inoculum size | 1 | 23.0535 | 23.0535 | 559.67 | 0.000 | 1 | 50.586 | 50.5856 | 317.90 | 0.000 |
| Culture volume | 1 | 0.0032 | 0.0032 | 0.08 | 0.784 | 1 | 2.501 | 2.5013 | 15.72 | 0.001 |
| Error | 18 | 0.7414 | 0.0412 |  |  | 18 | 2.864 | 0.1591 |  |  |
| Total | 26 | 83.1253 |  |  |  | 26 | 265.067 |  |  |  |
